# Human CPPED1 belongs to calcineurin‐like metallophosphoesterase superfamily and dephosphorylates PI3K‐AKT pathway component PAK4

**DOI:** 10.1111/jcmm.16607

**Published:** 2021-05-19

**Authors:** Antti M. Haapalainen, Ravindra Daddali, Mikko Hallman, Mika Rämet

**Affiliations:** ^1^ PEDEGO Research Unit and Medical Research Center Oulu University of Oulu Oulu Finland; ^2^ Department of Children and Adolescents Oulu University Hospital Oulu Finland; ^3^ Faculty of Medicine and Health Technology Tampere University Tampere Finland

**Keywords:** AKT, CPPED1, interactomics, microarray, PAK4, phosphatase, phosphatidylinositide 3‐kinase (PI 3‐kinase), PIK3R2, placenta, protein‐protein interactions

## Abstract

Protein kinases and phosphatases regulate cellular processes by reversible phosphorylation and dephosphorylation events. CPPED1 is a recently identified serine/threonine protein phosphatase that dephosphorylates AKT1 of the PI3K‐AKT signalling pathway. We previously showed that CPPED1 levels are down‐regulated in the human placenta during spontaneous term birth. In this study, based on sequence comparisons, we propose that CPPED1 is a member of the class III phosphodiesterase (PDE) subfamily within the calcineurin‐like metallophosphoesterase (MPE) superfamily rather than a member of the phosphoprotein phosphatase (PPP) or metal‐dependent protein phosphatase (PPM) protein families. We used a human proteome microarray to identify 36 proteins that putatively interact with CPPED1. Of these, GRB2, PAK4 and PIK3R2 are known to regulate the PI3K‐AKT pathway. We further confirmed CPPED1 interactions with PAK4 and PIK3R2 by coimmunoprecipitation analyses. We characterized the effect of CPPED1 on phosphorylation of PAK4 and PIK3R2 in vitro by mass spectrometry. CPPED1 dephosphorylated specific serine residues in PAK4, while phosphorylation levels in PIK3R2 remained unchanged. Our findings indicate that CPPED1 may regulate PI3K‐AKT pathway activity at multiple levels. Higher CPPED1 levels may inhibit PI3K‐AKT pathway maintaining pregnancy. Consequences of decreased CPPED1 expression during labour remain to be elucidated.

## 1 INTRODUCTION

Phosphates link to the hydroxyl groups of biomolecules via a phosphoester bond. Phosphoesters are also present in small molecules such as ATP, ADP and AMP.^1^ Protein kinases and phosphatases mediate reversible protein phosphorylation and dephosphorylation in signalling molecules and therefore are important for regulation of cellular events in all organisms. In eukaryotes, the hydroxyl groups of serine, threonine and tyrosine residues are the sites of phosphorylation and dephosphorylation. In the mammalian phosphoproteome, phosphoserine is the most prevalent of the three phosphoamino acids.^2^

Based on substrate specificity and catalytic mechanisms, protein phosphatases are classified as protein serine/threonine phosphatases (PSTPs or PSPs) or protein tyrosine phosphatases (PTPs). PSPs are further categorized into three classes: phosphoprotein phosphatases (PPPs), metal‐dependent protein phosphatases (PPMs) and aspartate‐based phosphatases.^3^ The PPP family is divided into PP1, PP2A, PP2B (also known as calcineurin and Ca^2+^ stimulated), PP4, PP5, PP6 and PP7 subclasses.^3^, ^4^ PPMs comprise protein phosphatases dependent upon Mn^2+^/Mg^2+^ ions for catalytic activity; PP2C is the largest PPM class. In aspartate‐based phosphatases, the catalytically important nucleophilic water molecule is replaced by the side chain of an aspartate residue.^5^ The catalytic subunits of several members of the PPP family associate with a wide variety of regulatory subunits.^3^, ^6^ By contrast, PPM members do not have regulatory subunits; instead, discrimination among substrates is achieved by the presence of additional domains and sequence motifs.

Based on the conserved fold, a vast number of phosphatases are members of the calcineurin‐like metallophosphoesterase (MPE) superfamily,^1^ and they may dephosphorylate lipids or nucleotides in addition to proteins. These phosphatases have a catalytic domain with a conserved calcineurin‐like fold, similar active‐site geometry and two sites for metal ion binding. The MPE superfamily consists of various types of phosphatases including pyrophosphatases, nucleases, phospholipases, purple acid phosphatases (PAPs), cyclic nucleotide phosphodiesterases (PDEs) and PPPs.^1^ MPEs catalyse the dephosphorylation reaction in a metal‐dependent manner. The presence of metal ions is critical, as they coordinate the catalytically important water molecule and position the phosphate of the substrate in the correct orientation in the active site. The crystal structures of MPE family members have revealed that various types of cations, such as Fe^2+^, Mn^2+^ and Zn^2+^, can reside in the active sites. One active site can contain two of the same cations or two different cations. For example, in PAPs, Fe^3+^ is the first cation of the metallic centre of the catalytic site, while the second metal can be Fe^2+^, Zn^2+^, Mn^2+^ or Mg^2+^.^7^, ^8^ Cyclic nucleotide PDEs hydrolyse both cAMPs and cGMPs, and class III PDEs can accept Fe^2+^, Mn^2+^ Mg^2+^, or Co^2+^ as catalysts.^9^

The phosphoinositide 3‐kinase (PI3K)‐AKT signalling pathway is evolutionarily conserved and is involved in cell metabolism, innate and adaptive immunity, and diseases such as cancer.^10^ Calcineurin‐like phosphoesterase domain‐containing protein 1 (CPPED1) dephosphorylates AKT1 at Ser473, thereby preventing cancer progression in bladder cancer.^11^ *CPPED1* expression levels are down‐regulated in non‐invasive bladder cancer tissue, whereas overexpression is associated with regression in tumour size. Additionally, CPPED1 regulates uptake of glucose in adipose tissue. Increased glucose uptake is associated with decreased *CPPED1* expression.^12^ Treatment with the PI3K‐specific inhibitor wortmannin decreases glucose uptake in *CPPED1* knockdown cells; this suggests that CPPED1 mediates glucose metabolism via the PI3K‐AKT signalling pathway.^12^ Moreover, silencing of *CPPED1* expression in the human trophoblast cell line HTR8/SVneo leads to up‐regulation of negative regulators of the PI3K pathway.^13^

In the present study, we used human proteome microarray technology to identify proteins that can interact with CPPED1 and to identify potential functions of CPPED1, particularly in the human placenta. A protein interactome study yielded a list of proteins, some of which have previously reported associations with the PI3K‐AKT signalling pathway; for example, p21 [RAC1] activated kinase 4 (PAK4) and phosphoinositide‐3‐kinase regulatory subunit 2 (PIK3R2). Coimmunoprecipitation (CoIP) and bimolecular fluorescence complementation (BiFC) methods revealed that CPPED1 interacts with PAK4 and PIK3R2 in vivo. Further, mass spectrometry analysis of CPPED1 with PAK4 and PIK3R2 revealed that CPPED1 dephosphorylated PAK4 at serine residues, whereas PIK3R2 phosphorylation levels remained unaffected. These findings suggest that CPPED1 not only inhibits the activity of AKT1 but also affects the function of PI3K. We also show that CPPED1 mediates the effect of progesterone on the expression of certain genes in human placental HTR8/SVneo trophoblast cells.

## 2 MATERIALS AND METHODS

### 2.1 Homology search and sequence alignment

We used the human CPPED1 amino acid sequence and Network Protein Sequence Analysis (NPS@) software ^14^ (https://prabi.ibcp.fr/htm/site/web/home) to search for similar proteins in the SwissProt database. Similar sequences were aligned, and sequence similarity values were obtained with GONNET protein weight matrix in the Clustalw tool in NPS@. Graphics of the aligned sequences were modified in ESPript 3.0.^15^

### 2.2 Construction of plasmids for protein microarray, coimmunoprecipitation and bimolecular fluorescence complementation experiments

Primers used for cloning into different plasmids are listed in Table S1. pBiFC‐VN173 (Addgene plasmid #22010) and pBiFC‐VC155 (Addgene plasmid #22011) were gifts from Chang‐Deng Hu.^16^ Further details are presented in the Supporting Information.

### 2.3 Recombinant CPPED1 expression and purification

CPPED1 cloned into the PSF‐OXB20‐NH2‐6HIS‐V5‐TEV constitutive expression plasmid was transformed and expressed in *E coli* BL21(DE3) cells at 37°C for 7‐8 h. Cells were then harvested. After cell lysis by sonication, most of the CPPED1 was in a soluble form. CPPED1 was purified with Ni‐NTA affinity column, hydrophobic interaction chromatography (HIC) Resource ISO column and size‐exclusion HiLoad 16/60 Superdex 200 column (Fig. S1). Further details are presented in the Supporting Information.

### 2.4 Identification of recombinant CPPED1 by western blot

CPPED1 was expressed as a recombinant protein with an N‐terminal His‐V5 dual tag for protein microarray experiments. To confirm that the purified protein was CPPED1 and had the V5 tag in the N terminus, we used anti‐CPPED1 and anti‐V5 antibodies (Fig. S2). Further details are presented in the Supporting Information.

### 2.5 Detection of CPPED1 phosphatase activity by western blot

**FIGURE 1:**
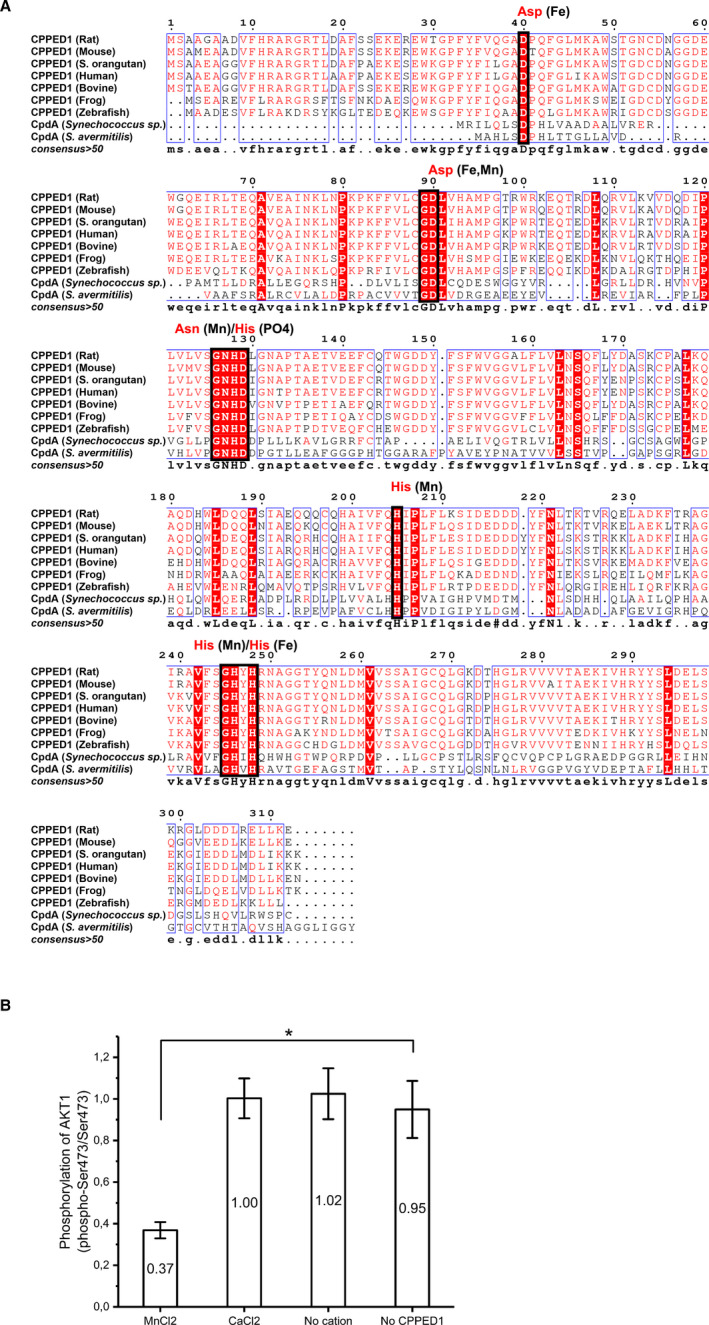
Alignment of CPPED1 with 3′,5′‐cyclic adenosine monophosphate phosphodiesterase, and in vitro phosphatase activity of CPPED1. (A) CPPED1 homologous sequences were searched with NPS@ software.^14^ CPPED1 showed similarities with 3′,5′‐cyclic adenosine monophosphate phosphodiesterase (CpdA). Seven eukaryotic CPPED1 sequences are aligned with the two closest CpdA homologs from *Synechococcus sp* and *Streptomyces avermitilis*. Amino acid residues conserved throughout the sequences are highlighted in red. Similar residues are denoted by red letters. Residues that belong to the class III cAMP phosphodiesterase (PDE) sequence motif are framed. Framed residues are involved in metal coordination or phosphate binding. Amino acid residue and the interacting group (Fe^3+^, Mn^2+^ or PO_4_) are shown above the sequence alignment. These interactions were obtained from the crystal structure of *Mycobacterium tuberculosis* PDE (Protein Data Bank code 3IB8) and Matange.^9^ Conserved class III cAMP PDE motif: D‐[X]_n_‐GD‐[X]_n_‐GNH[E/D]‐[X]_n_‐H‐[X]_n_‐GHXH. Figure prepared with ESPript 3.0.^15^ (B) Phosphorylation of Ser473 of AKT1 was quantified and compared with total AKT1 with and without addition of CPPED1. Effects of Mn^2+^ and Ca^2+^ on CPPED1 activity were also tested. Reaction mixture consisting of AKT1 alone was used as the reference to which other reaction mixtures were compared. pS473/S473 ratio values are shown. Significant differences were estimated with the nonparametric Mann‐Whitney *U* test (n = 6 per group). Statistically significant change is indicated by an asterisk. (*P* <.05)

Previous studies have shown that CPPED1 dephosphorylates AKT1.^11^ To confirm that our purified human recombinant CPPED1 was active, we followed the in vitro phosphatase assay protocol of Gao et al.^17^ Dephosphorylation reactions were carried out in a reaction buffer that contained 50 mM Tris (pH 7.4), 1 mM dithiothreitol and 5 mM MnCl_2_ or CaCl_2_ at 30°C for 30 min (Figure 1B). The final concentration of CPPED1 and AKT1 (009‐001‐P21, Rockland) in the reactions was 0.1 µM and 0.13 µM, respectively. Significant differences were estimated with the nonparametric Mann‐Whitney *U* test (n = 3 per sample type). Further details are presented in the Supporting Information.

### 2.6 Circular dichroism and static light scattering

CD spectroscopy was performed with a Chirascan CD spectrometer (Applied Photophysics). CD data were collected between 280 and 200 nm at 22°C with a 0.1‐cm path length quartz cuvette (Fig. S3). The Superdex 200 HR 5/150 GL (GE Healthcare) size‐exclusion column and SLS instrument (Wyatt Technology) were used to determine the monodispersity of the purified recombinant human CPPED1 (Fig. S4). Further details are presented in the Supporting Information.

### 2.7 Human proteome microarray

The HuProt^TM^ v3.1‐Human Proteome Microarray (Cambridge Protein Arrays Ltd.) was used to identify protein interactions on an immobilized array. The protein microarray contains >20 000 human recombinant proteins and covers approximately 75% of the annotated protein‐coding genome. Recombinant proteins on the microarray were expressed in *Saccharomyces cerevisiae* and purified with the GST tag purification system. Further details are presented in the Supporting Information.

### 2.8 Fluorescence colocalization analysis

**FIGURE 2:**
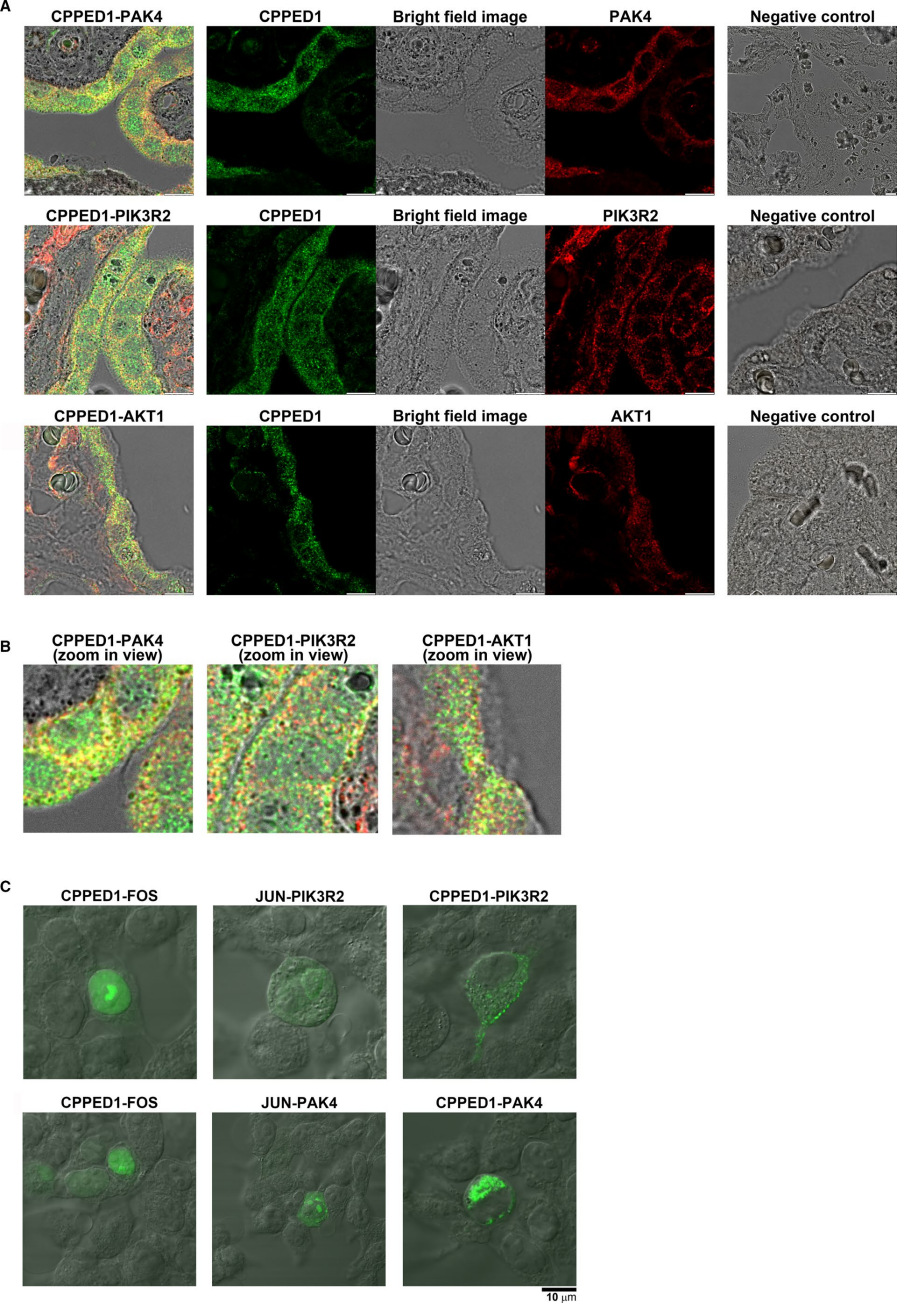
(A) Confocal microscopy images of human placenta expressing endogenous CPPED1, PAK4, PIK3R2 and AKT1. Colocalization fluorescence image of CPPED1 (green) and PAK4 (red) (top row), CPPED1 (green) and (PIK3R2) (red) (middle row), and CPPED1 (green) and AKT1 (red) (bottom row). Middle panel shows green channel, red channel and bright field image separately of the left panel. Panels on the right show negative controls treated the same way as samples but with primary antibody omitted. Objective used was HC PL APO 63×/1.40 OIL CS2 DIC (oil). Scale bar represents 10 µm. (B) Zoom in view of the images shown in A (left panels). (C) Confocal microscopy images of transfected HEK293T cells coexpressing CPPED1 with PIK3R2 and PAK4. Fluorescence image of HEK293T cells coexpressing CPPED1‐VN and PIK3R2‐VC (top row, right panel) and CPPED1‐VN and PAK4‐VC (bottom row, right panel). VN is the N‐terminal fragment of Venus yellow fluorescence protein (YFP), and VC is the C‐terminal fragment of Venus YFP. CPPED1 or JUN (control) was fused to the N‐terminal fragment of Venus, while PIK3R2, PAK4 or FOS (control) was fused to the C‐terminal fragment of Venus. If the two coexpressing proteins interact, N‐ and C‐terminal fragments of Venus YFP are brought together, which results in stable intact Venus YFP and a fluorescence signal. As controls, CPPED1‐VN and FOS‐VC (top row, left panel) and JUN‐VN and PIK3R2‐VC (top row, middle panel) were coexpressed in HEK293T cells. Similarly, JUN‐VN and PAK4‐VC (bottom row, middle panel) were coexpressed in HEK293T cells. Objective: UPLSAPO 60×/1.35 (oil). Scale bar same for all images

Fluorescence colocalization analysis was performed with trophoblast cell and human placenta tissue samples. HTR8/SVneo cells were seeded into the CELLview Cell Culture Dish (35 mm; Greiner Bio‐One), which has a glass bottom, and the cells were grown as described previously.^13^ Immunofluorescence staining on cell culture dishes was done as described previously,^18^ using 0.1% saponin for cell permeabilization. Human placental tissue samples were treated as described previously.^13^ Colocalization of the following complexes was analysed: CPPED1‐AKT1, CPPED1‐PAK4 and CPPED1‐PIK3R2 (Figure 2A,B, Fig. S5). Antibodies and detection methods are presented in the Supporting Information.

### 2.9 Bimolecular fluorescence complementation assay

HEK293T cells were seeded into the CELLview Cell Culture Dish (35 mm; Greiner Bio‐One), which has a glass bottom. Cells were grown and transfected as described earlier for the CoIP experiments. In the controls, 125 ng of CPPED1‐VN, JUN‐VN (pBiFC‐bJunVN173, Addgene plasmid #22012), PAK4‐VC, PIK3R2‐VC or FOS‐VC (pBiFC‐bFosVC155, Addgene plasmid #22013) plasmid were transfected into cells (Fig. S6). For the BiFC assay, the following combinations of two plasmids (30 ng each) were transfected into HEK293T cells: CPPED1‐VN and PIK3R2‐VC, CPPED1‐VN and PAK4‐VC, CPPED1‐VN and FOS‐VC, JUN‐VN and PIK3R2‐VC, JUN‐VN and PAK4‐VC, and JUN‐VN and FOS‐VC (Figure 2C, Fig. S6). The detection was done as presented in the Supporting Information.

### 2.10 Coimmunoprecipitation

HEK293T cells were seeded into 60‐mm dishes and grown overnight in DMEM (Invitrogen Life Technologies). Cells were transfected with either CPPED1‐pCMV‐Flag 2B (3 µg) and PAK4‐pcDNA3‐myc (3 µg) plasmids or CPPED1‐pCMV‐Flag 2B (3 µg) and PIK3R2‐pcDNA3‐myc (3 µg) plasmids and then incubated for 48 h. We performed three independent experiments for both transfections. Cells were lysed in the presence of protease inhibitors (Halt™ Protease Inhibitor Cocktail, Thermo Fisher Scientific). Cell lysis, pre‐clearing and CoIP were performed in accordance with the manufacturer's instructions (Pierce^™^ Co‐Immunoprecipitation Kit, Thermo Fisher Scientific). CoIP was done with anti‐c‐Myc agarose (PierceTM Anti‐c‐Myc Agarose, Thermo Fisher Scientific). Prior to CoIP, both anti‐c‐Myc agarose and control agarose resins were blocked with 1% BSA at 4°C for 1 h.

CoIP samples were separated by SDS‐PAGE and immunoblotted as described previously.^13^, ^19^ The detection was done as presented in the Supporting Information.

### 2.11 Phosphatase assay detection by mass spectrometry

In vitro dephosphorylation reactions with CPPED1, PAK4 and PIK3R2 were performed as described earlier for AKT1,^17^ except that dephosphorylation of PAK4 and PIK3R2 was detected by mass spectrometry after addition of 5 mM MnCl_2_, MgCl_2_ or CaCl_2_. In the reaction mixture (50 µl), the final concentrations of CPPED1 (purified by us), PAK4 (TP302302; Origene) and PIK3R2 (009‐001‐S95S; Rockland) were 0.13 µM, 0.16 µM and 0.15 µM, respectively. After the phosphatase assay, the samples were treated and measured as presented in the Supporting Information.

### 2.12 Transfection of small interfering RNAs and progesterone treatment of transfected HTR8/SVneo cells

HTR8/SVneo cells (ATCC, CRL‐3271) were transfected with either negative control siRNA (siNEG, 30 nM) or pooled *CPPED1* siRNAs (siCPPED1, 30 nM each siRNA for pairs 1‐3), as described previously.^13^ After the transfection experiment, cells were harvested and RNA isolated. Transcriptomes of samples were determined with the Illumina HiSeq high‐throughput sequencing system at the Finnish Functional Genomics Centre, Finland. Sequencing data were analysed by the Bioinformatics Unit Core Service at the Turku Centre for Biotechnology, Finland. Further details are presented in the Supporting Information.

## 3 RESULTS

### 3.1 Sequence homology places CPPED1 in the cAMP phosphodiesterase family

CPPED1 has a calcineurin‐like phosphoesterase domain and is inhibited by trifluoperazine, which suggests that CPPED1 could belong to the PP2A or PP2B families of PPPs.^11^ This classification prompted us to perform sequence homology searches with human CPPED1. We did not detect the conserved amino acid sequence motifs of PPP and PPM family members in the CPPED1 sequence.^3^ Moreover, CPPED1 lacked the two active‐site loops of PPP and PPM family members.^20^ Our homology search indicated that the two closest homologs of human CPPED1 are 3′,5′‐cyclic adenosine monophosphate (cAMP) phosphodiesterase (PDE) (CpdA) from *Synechococcus sp* (Figure 1A) and purple acid phosphatase 22 (PAP22) from *Arabidopsis thaliana* (Fig. S7), with 47% and 42% sequence similarity, respectively. Concerning PAPs, CPPED1 is missing the three conserved and functionally important amino acid residues of the PAP active site (Fig. S7). These three residues are important for Fe (III) coordination and phosphate binding.^21^ In particular, the tyrosine that is replaced by histidine in CPPED1 is important for Fe (III) binding; this tyrosinate‐Fe (III) charge transfer results in the purple colour at 560 nm. Instead, we found that CPPED1 has the characteristic sequence motif of the class III cAMP PDE family (Figure 1A). Class III cAMP PDEs belong to a family of calcineurin‐like metallophosphoesterases (MPE).^1^ These findings suggest that human CPPED1 belongs to the class III PDE of MPE superfamily rather than to the PPP or PPM families.

### 3.2 Purification and characterization of human recombinant CPPED1 for human proteome microarray

To overexpress and purify recombinant CPPED1 protein, we cloned *CPPED1* into the constitutively active expression plasmid pSFOXB20 and expressed the recombinant protein with the N‐terminal His‐V5 dual tag in *E coli* BL21 (DE3) cells. CPPED1 was expressed as the soluble form and purified to apparent homogeneity by affinity, hydrophobic interaction and size‐exclusion chromatography. Protein purity was analysed by SDS‐gel electrophoresis (Fig. S1).

To ensure that the purified recombinant CPPED1 was folded properly, monodispersed and enzymatically active, we carried out circular dichroism (CD) spectroscopy, static light scattering (SLS) and in vitro activity measurements, respectively. CD spectroscopy confirmed that human recombinant CPPED1 was folded properly (Fig. S3) and comprised both α‐helical and β‐sheet structures. Based on the amino acid sequence including the His‐V5 dual tag, the size of the human recombinant CPPED1 was 38.9 kDa. SLS measurements indicated that the purified CPPED1 protein sample was homogenous (Fig. S4) and monomeric.

CPPED1 was previously shown to dephosphorylate phospho‐Ser473 of AKT1.^11^, ^17^ To confirm that our purified recombinant CPPED1 was enzymatically active, we carried out in vitro phosphatase assays with Mn^2+^ in accordance with the methods of Gao et al.^17^ We also incubated CPPED1 and AKT1 together in reaction buffer with Ca^2+^ and without cations (Mn^2+^ or Ca^2+^) to determine whether CPPED1‐mediated dephosphorylation is cation dependent. Ca^2+^ was added because CPPED1 was observed to have PP2B‐like protein phosphatase activity,^11^ and PP2B‐like phosphatases are stimulated by Ca^2+^.^3^, ^4^ CPPED1 phosphatase activity was cation dependent and showed higher enzymatic activity in the presence of Mn^2+^ (Figure 1B). Once we had confirmed that purified recombinant CPPED1 was folded properly, enzymatically active and monomeric in solution, we used it in the HuProt^TM^ human proteome microarray chip platform to identify target proteins for CPPED1.

### 3.3 Identification of human CPPED1‐interacting proteins by protein microarray

Protein microarrays can be used to identify protein‐protein interactions, posttranslational protein modifications and modulations in protein expression levels under different conditions. To identify novel targets for CPPED1, we used the HuProt^™^ v3.1 human proteome microarray to screen for protein‐protein interactions with CPPED1. The HuProt^™^ v3.1 microarray covers approximately 75% of the annotated human protein‐coding genome. Analysis yielded a list of 36 proteins (Table 1) that interact with CPPED1 in vitro. Although CPPED1 has been shown to dephosphorylate AKT1,^11^ AKT1 was not in the list of CPPED1 interacting proteins. We then performed a DAVID functional annotation study that included all of the identified binding partners of CPPED1 (Table 2); this study identified growth factor receptor bound protein 2 (GRB2), PAK4 and PIK3R2 as enriched in the pathway analysis. In a previous study, we showed that knockdown of *CPPED1* in HTR8/SVneo trophoblasts leads to enhanced expression of negative regulatory genes of the PI3K pathway, such as phosphoinositide‐3‐kinase interacting protein 1 (*PIK3IP1*) and phosphatidylinositol‐4,5‐bisphosphate 3‐kinase catalytic subunit gamma (*PIK3CG*).^13^ PAK4 binds to phosphoinositide‐3‐kinase regulatory subunit 1 (PIK3R1)^22^ and activates the PI3K pathway, whereas CPPED1, according to our protein microarray analysis, bound to PIK3R2 and PAK4. Based on these findings, we continued to investigate interactions between CPPED1 and PAK4 and PIK3R2.

**TABLE 1:** CPPED1‐interacting proteins obtained by human proteome microarray. Above‐threshold interactions are listed in order of highest to lowest binding affinity with CPPED1

Target symbol	Target name
C1QTNF5	C1q and TNF related 5
DDX6	DEAD‐box helicase 6
SORBS1	sorbin and SH3 domain containing 1
WIPF1	WAS/WASL interacting protein family member 1
CRK	CRK proto‐oncogene, adaptor protein
POGZ	pogo transposable element derived with ZNF domain
RBM42	RNA binding motif protein 42
PAK4	p21 (RAC1) activated kinase 4
GRB2	growth factor receptor bound protein 2
VCL	Vinculin
KCNAB2	potassium voltage‐gated channel subfamily A regulatory beta subunit 2
QKI	QKI, KH domain‐containing RNA binding
CDCA3	Cell division cycle associated 3
PNKP	Polynucleotide kinase 3'‐phosphatase
MBP	Myelin basic protein
ISG20	Interferon‐stimulated exonuclease gene 20
ADAT3	Adenosine deaminase, tRNA specific 3
SF3B4	Splicing factor 3b subunit 4
NAT6	N‐acetyltransferase 6
PIK3R2	Phosphoinositide‐3‐kinase regulatory subunit 2
API5	Apoptosis inhibitor 5
RTCA	RNA 3'‐terminal phosphate cyclase
WWP2	WW domain‐containing E3 ubiquitin‐protein ligase 2
CXCL16	C‐X‐C motif chemokine ligand 16
IRF2BP2	Interferon regulatory factor 2 binding protein 2
ACOX1	Acyl‐CoA oxidase 1
KHDRBS1	KH RNA binding domain containing, signal transduction associated 1
COASY	Coenzyme A synthase
HNRNPK	Heterogeneous nuclear ribonucleoprotein K
HNRNPD	Heterogeneous nuclear ribonucleoprotein D
KLHDC9	Kelch domain containing 9
ZNF207	Zinc finger protein 207
ANXA11	Annexin A11
LAPTM4A	Lysosomal protein transmembrane 4 alpha
SMARCC1	SWI/SNF‐related, matrix‐associated, actin‐dependent regulator of chromatin subfamily c member 1
HTATIP2	HIV‐1 Tat interactive protein 2

**TABLE 2:** KEGG pathway analysis of CPPED1 binding partners identified by human proteome microarray. Significant terms (*P* <.05) are shown

Term	Genes	*p* Value	Benjamini *p* value
hsa05211:Renal cell carcinoma	*CRK, GRB2, PAK4, PIK3R2*	0.000285	0.025583
hsa04510:Focal adhesion	*CRK, GRB2, PAK4, PIK3R2, VCL*	0.000617	0.02771
hsa04012:ErbB signalling pathway	*CRK, GRB2, PAK4 PIK3R2*	0.000643	0.029325
hsa04910:Insulin signalling pathway	*CRK2,GRB2,PIK3R2, SORBS1*	0.002445	0.054172
hsa04062:Chemokine signalling pathway	*CXCL16, CRK, GRB2, PIK3R2*	0.005682	0.098513
hsa04810:Regulation of actin cytoskeleton	*CRK, PAK4,PIK3R2, VCL*	0.007959	0.114135
hsa05220:Chronic myeloid leukaemia	*CRK, GRB2, PIK3R2*	0.009064	0.111629
hsa05100:Bacterial invasion of epithelial cells	*CRK, PIK3R2, VCL*	0.010575	0.113904
hsa04660:T cell receptor signalling pathway	*GRB2, PAK4, PIK3R2*	0.016992	0.159101
hsa05206:MicroRNAs in cancer	*CRK, GRB2, HNRNPK, PAK4*	0.018409	0.155558
hsa04722:Neurotrophin signalling pathway	*CRK, GRB2, PIK3R2*	0.023948	0.181701

### 3.4 Subcellular colocalization of CPPED1‐PAK4, CPPED1‐PIK3R2 and CPPED1‐AKT1 pairs

Protein microarray experiment results showed that CPPED1 bound to both PAK4 and PIK3R2 in vitro. Next, we explored whether PAK4 and PIK3R2 colocalize with CPPED1 in cells. We also investigated colocalization of CPPED1 and AKT1. In the colocalization experiment, we observed endogenous proteins in both HTR8/SVneo trophoblast cells (Fig. S5) and placental tissue samples (Figure 2A,B). In HTR8/SVneo cells, CPPED1, PAK4, PIK3R2 and AKT1 colocalized. However, the AKT1 distribution pattern was slightly different from those of CPPED1, PAK4 and PIK3R2. Namely, the signal from AKT1 mostly originated from the network structure within cells (Fig. S5C). By contrast, CPPED1, PAK4 and PIK3R2 localization resembled individual spot patterns (Fig. S5A, Fig. S5B). In placental tissue samples, CPPED1, PAK4, PIK3R2 and AKT1 all localized in the cytosol. However, CPPED1 and PIK3R2 were found more on the cell membranes, and CPPED1 and PAK4 were found more around nucleus and close to cell membrane (Figure 2A,B).

Next, we visualized protein‐protein interactions within a cell by bimolecular fluorescence complementation (BiFC). In BiFC, proteins fused to the N‐ and C‐terminal fragments of yellow fluorescent protein (YFP) or variants^23^, ^24^ are coexpressed. Protein‐protein interactions between the two putative protein‐binding partners bring together the two halves of YFP, which results in a fluorescence signal. BiFC is used to investigate interactions between proteins in a cellular milieu as well as subcellular localization.^16^, ^25^

We used BiFC to verify interactions between CPPED1 and PAK4 and PIK3R2 and to determine the subcellular localization of the protein complex. For the interaction analysis, we cloned CPPED1 into the N‐terminal region of pBiFC‐VN173 (CPPED1‐VN), and PAK4 and PIK3R2 into the C‐terminal region of pBiFC‐VC155 (PAK4‐VC and PIK3R2‐VC, respectively). As a positive control, we used JUN‐VN and FOS‐VC plasmids. Fos and Jun are transcription factors that form heterodimers in the nucleus.^26^

When single plasmids, either VN or VC variants, were transfected into HEK293T cells, we did not observe a fluorescence signal (Fig. S6A,B). After cotransfection with JUN‐VN and FOS‐VC, there was a strong fluorescence signal in the nucleus, which confirmed the validity of the method for the identification of protein interactions and subcellular localization of protein complexes (Fig. S6C). Cells expressing CPPED1 and PIK3R2 showed a fluorescence signal in the cytosol, with high‐intensity spots at the cell membrane (Figure 2C, right panel). However, control experiments performed with CPPED1‐VN and FOS‐VC (Figure 2C, left panel) or JUN‐VN and PIK3R2‐VC (Figure 2C, middle panel) also resulted in fluorescence signal. The subcellular localization of the CPPED1–FOS and JUN‐PIK3R2 complexes was different, however. Whereas the CPPED1‐FOS complex was solely in the nucleus (Figure 2C, left panel), the CPPED1‐PIK3R2 complex was found at the cytoplasmic side of the cell membrane (Figure 2C, right panel). Similarly, even though the control experiment, JUN‐VN and PAK4‐VC (Figure 2C, middle panel) resulted in fluorescence signal, the fluorescence signal of CPPED1‐VN and PAK‐VC showed different type of fluorescence patter in the cytosol (Figure 2B, right panel).

We conclude that this method could not be used to demonstrate whether two proteins interact under normal conditions, as it appears to be too sensitive; when highly expressed, the YFP halves have a tendency to self‐assemble, resulting in a stable YFP independent of protein‐protein interactions between the two proteins under investigation.^23^, ^24^ However, it is feasible to use BiFC to investigate the subcellular localization of known interactions. In summary, in our experimental setting, the BiFC assay could not confirm interactions between CPPED1 and its putative binding partners PAK4 and PIK3R2. However, it did indicate that CPPED1‐PIK3R2 and CPPED1‐PAK4 (Figure 2C) complexes were cell membrane associated and cytosolic, respectively.

### 3.5 Coimmunoprecipitation of PAK4 and PIK3R2 with CPPED1

**FIGURE 3:**
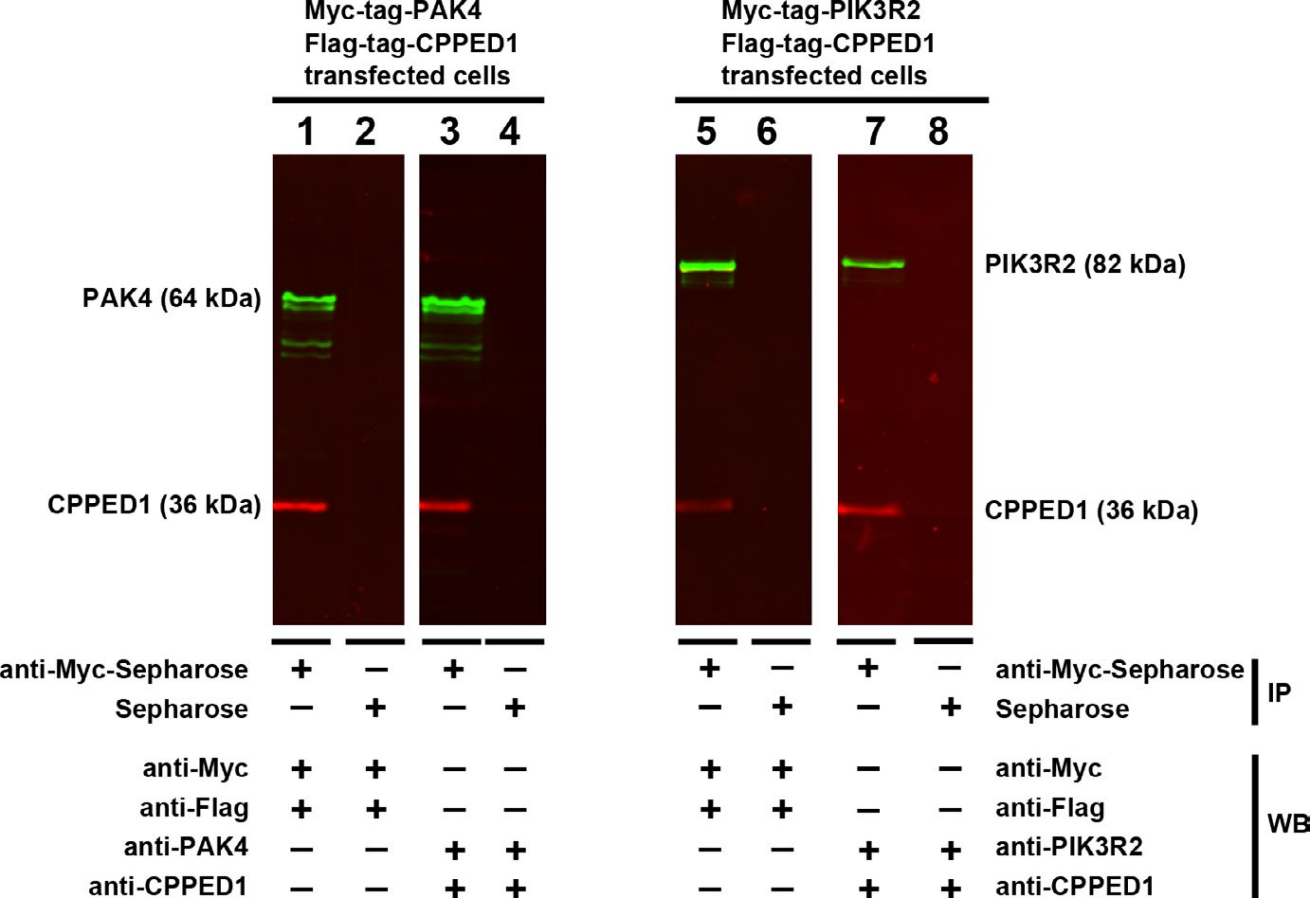
Coimmunoprecipitation of CPPED1, PAK4 and PIK3R2. Myc‐tagged PAK4 and flag‐tagged CPPED1 (left panel) and myc‐tagged PIK3R2 and flag‐tagged CPPED1 (right panel) were transiently expressed in HEK293T cells. Tagged proteins PAK4 and PIK3R2 were coimmunoprecipitated with anti‐myc Sepharose. Except for the negative control, in which anti‐myc Sepharose was replaced by Sepharose, all samples were treated in the same manner. Immunocomplexes were analysed by Western blot (WB), and by using different antibodies for the same immunoprecipitation (IP) sample. Antibodies used were anti‐myc, anti‐flag anti‐CPPED1, anti‐PAK4 and anti‐PIK3R2. The following IP samples are the same: 1 and 3, 2 and 4, 5 and 7, and 6 and 8. For example, in the Western blot, anti‐myc and anti‐flag antibodies were used with sample 1, and anti‐PAK4 and anti‐CPPED1 antibodies were used with sample 3. The original blots are presented in Figs. S8 and S9

Since PAK4 and PIK3R2 colocalized with CPPED1 in cells, we carried out CoIP analysis to investigate whether these protein complexes formed in vivo. HEK293T cells were cotransfected with plasmids expressing CPPED1 and PAK4 or CPPED1 and PIK3R2. PAK4 or PIK3R2 were expressed as recombinant proteins with an N‐terminal myc‐tag, and anti‐myc antibody was used for the pulldown; afterwards, protein complexes were analysed by Western blot. Successful pulldown of the bait was verified with anti‐myc, anti‐PAK4, or anti‐PIK3R2 antibodies (Figure 3, Fig. S8, Fig. S9). Anti‐flag and anti‐CPPED1 antibodies were used to detect CPPED1 in the complexes. Overexpressed CPPED1 coimmunoprecipitated with PAK4 or PIK3R2 (Figure 3, Fig. S8, Fig. S9). These results provided further evidence that CPPED1 interacts with PAK4 and PIK3R2.

### 3.6 CPPED1 dephosphorylates PAK4 in vitro

**FIGURE 4:**
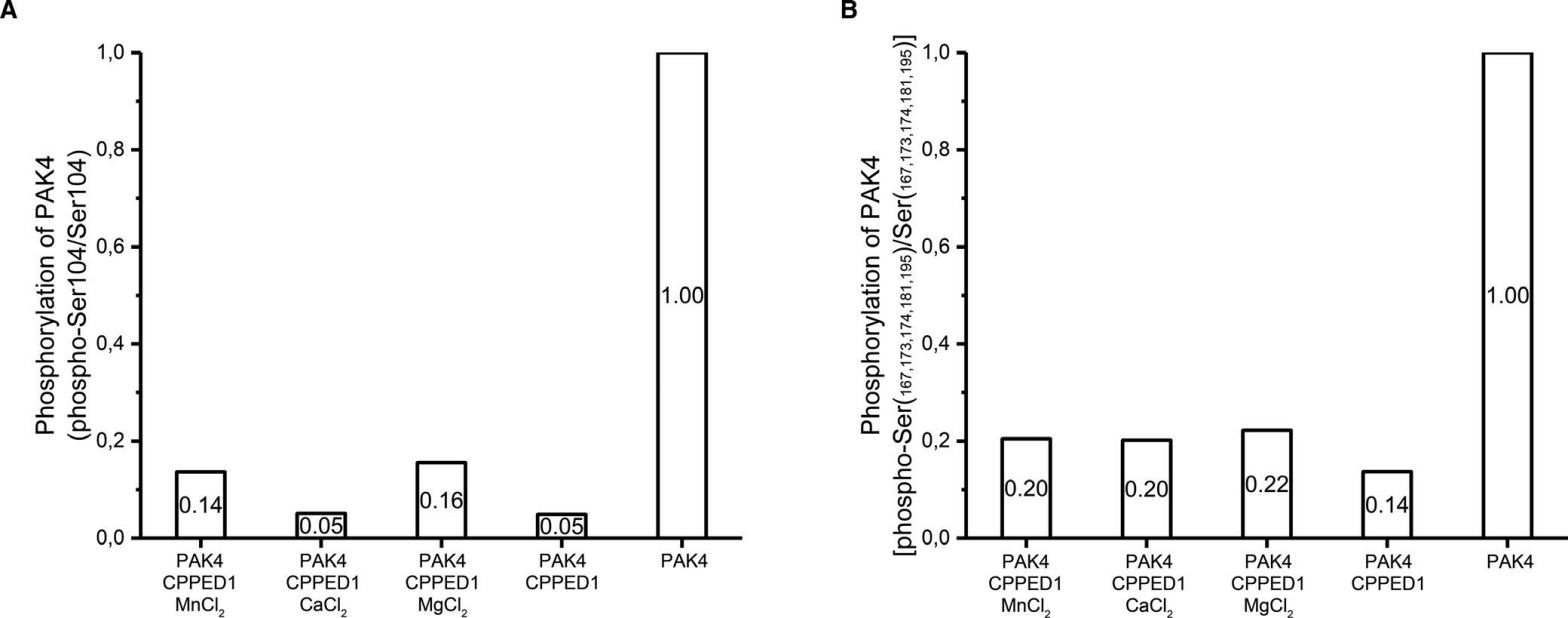
In vitro phosphatase activity of CPPED1 determined by mass spectrometry. Human recombinant PAK4 incubated with and without human recombinant CPPED1. Effect of cations Mn^2+^, Ca^2+^ or Mg^2+^ on phosphatase activity was also measured. Phosphopeptides of PAK4 determined by mass spectrometry. Two phosphopeptides showed a decrease in their phosphorylation content. The changes were seen at Ser104 (A) and Ser167, Ser173, Ser174, Ser181 and Ser195 (B)

CPPED1 is known to dephosphorylate AKT1.^11^ According to the SwissProt database, PAK4 and PIK3R2 are phosphorylated at various sites including serine and threonine residues. We investigated whether CPPED1 dephosphorylates any of the phosphorylated residues of PAK4 and PIK3R2. To do so, we incubated CPPED1 with PAK4 or PIK3R2 and looked for changes in the phosphorylated residues by mass spectrometry. We detected numerous phosphorylated serines, threonines, and tyrosines in PAK4 (Fig. S10) and PIK3R2 (Fig. S11). None of the identified phosphorylations decreased in PIK3R2 in the presence of CPPED1. In the PAK4 assay, there were two phosphopeptides in which the number of phosphoserines decreased when CPPED1 was included in the reaction mixture (Figure 4). Phosphorylation at Ser104 of the Ser97‐Arg110 peptide decreased 85%‐95%. The other phosphopeptide, Glu165‐Lys198, contained five phosphoserines; phosphorylation of this peptide decreased 80‐85% in the presence of CPPED1. We could not determine the specific dephosphorylation sites with this method; thus, we are uncertain whether CPPED1 dephosphorylates all or most of sites Ser167, Ser173, Ser174, Ser181 and Ser195 in vitro. Furthermore, addition of cations (Mn^2+^, Ca^2+^, Mg^2+^) was not required for the phosphatase activity of CPEDD1 in this setting. In summary, while CPPED1 dephosphorylated PAK4 at several serines in vitro, we were unable to detect any CPPED1 phosphatase activity upon PIK3R2.

### 3.7 CPPED1 mediates the effect of progesterone on gene expression in HTR8/SVneo cells

To determine whether CPPED1 mediates the effect of progesterone on gene expression, we treated *CPPED1*‐silenced and *CPPED1*‐unsilenced HTR8/SVneo cells with progesterone (P4). Silencing was accomplished with small interfering RNA (siRNA), as described previously.^13^ Based on qRT‐PCR results, silencing of *CPPED1* at the mRNA level was 68%.^13^ When gene expression levels of HTR‐8/SVneo cells were analysed by RNA sequencing, expression levels of 98 genes were either down (n = 46; Table S2) or up (n = 52; Table S3) in P4‐treated vs. untreated control cells. When we compared this gene list with the transcriptomes of cells in which there was simultaneous silencing of *CPPED1* expression and P4 treatment (Table S4), we found that silencing of *CPPED1* expression removed the effect of P4 on the expression of a subset of genes (n = 71; Table S4, Fig. S12). Out of these 71 genes, the expression levels of 36 genes change more than 50% after silencing of *CPPED1* expression (Table S4). This suggests that in HTR8/SVneo cells, CPPED1 mediates the effect of P4 on the expression of certain genes.

## 4 DISCUSSION

A previous report placed CPPED1 in both the PP2A and PP2B subclasses of the PPP protein family.^11^ Based on the amino acid sequence, CPPED1 is similar to PP2A protein phosphatases, while CPPED1 phosphatase activity is inhibited by trifluoperazine, which suggests that CPPED1 is a PP2B protein. Our thorough comparison of the CPPED1 sequence with PPP family members revealed that certain conserved signature motif sequences associated with PPPs, such as GDxHG (for metal ion binding), GDxVDRG and HGG (for both metal binding and phosphate binding) sequences, were absent in CPPED1. Moreover, the GNHE motif of PPPs was replaced by GNHD in CPPED1. Similarly, the RxxxD, DGxxG, DG and GxxDN motifs of PPMs were absent in CPPED1. Despite its overall sequence similarity with PP2A proteins, CPPED1 may belong to neither the PPP nor the PPM protein families. This prompted us to further investigate the function of CPPED1 by sequence homology searches and interactomics.

Homology and sequence alignment studies revealed that the closest homologs of CPPED1 were PAP and cAMP PDE from plant and bacteria, respectively. PAPs are a group of nonspecific phosphomonoesterases found in animals, plants and fungi. Mammalian PAPs are highly conserved binuclear metal‐containing enzymes that are produced by osteoclasts and involved in bone resorption. Features of PAPs include formation of a purple colour in concentrated solutions and resistance to inhibition by L‐tartrate.^27^ However, as with PPPs and PPMs, CPPED1 lacks some of the key residues of the PAP family. These residues are important for Fe (III) coordination, phosphate binding, and, especially, the tyrosinate‐Fe (III) charge transfer that results in a purple colour.^21^ This suggests that CPPED1 is not a PAP. The other close homolog of CPPED1, cAMP PDE, hydrolyses cAMP and negatively regulates cyclic nucleotide–mediated signal transduction pathways. Cyclic nucleotide PDEs are commonly divided into three classes based on their sequence homology.^9^ Class III PDEs are widespread phylogenetically and are found in eukaryotes, archaea and bacteria, and they contain the highly conserved signature motif D‐[x]n‐GD‐[x]n‐GNH[E/D]‐[x]n‐H‐[x]n‐GHxH. We found that human CPPED1 had the complete class III PDE signature motif and that the closet homolog of CPPED1 was a class III PDE from bacteria. The class III PDE family is part of the calcineurin‐like MPE superfamily. Members of the MPE superfamily share a conserved core MPE fold called the calcineurin‐like fold, which resembles calcineurin protein phosphatase.^1^ Based on these findings, it is likely that CPPED1 belongs to the class III PDE family of the MPE superfamily and shares similar structural and catalytic properties.

CPPED1 dephosphorylates AKT1 in the presence of cation.^11^, ^17^ Similarly, we observed that CPPED1 requires cations for dephosphorylation of AKT, with Mn^2+^ serving as a better catalyst than Ca^2+^. As mentioned previously, CPPED1 presumably belongs to the class III PDE protein family of the MPE superfamily. Class III PDEs bind two divalent cations in the active site, and the most prevalent catalytic metals are Fe^2+^ and Mn^2+^.^9^ While class III PDEs accept cyclic nucleotides and the phosphomonoesters AMP and ATP as substrates, AKT1, the substrate of CPPED1, is a protein. However, class III PDEs are inhibited by phosphoserines.^28^ This suggests that class III PDEs such as CPPED1 could accept the phosphoserine of a protein as a substrate, provided the surfaces of the binding partners favour protein‐protein interactions. Furthermore, the active‐site residues and catalytically important metal ions are located on the surface of class III PDE proteins (eg PDB code 1S3N), which may facilitate catalysis of phosphoproteins.^1^

We used a human proteome microarray to identify the putative in vivo binding partners of CPPED1. Out of 19,000 proteins, CPPED1 interacted with 36 proteins. Coimmunoprecipitation and BiFC studies confirmed the interaction between CPPED1 and PAK4 as well as between CPPED1 and PIK3R2. PAK4 and PIK3R2 are known to regulate the PI3K‐AKT pathway.^22^, ^29^, ^30^ Moreover, our previous studies revealed that knockdown of *CPPED1* expression leads to up‐regulation of negative regulatory proteins of the PI3K‐AKT pathway, such as PIK3IP1 ^31^ and insulin‐like growth factor‐binding protein 5 (IGFBP5).^13^, ^32^ Furthermore, inositol polyphosphate‐5‐phosphatase D (INPP5D) was down‐regulated. INPP5D converts phosphatidylinositol (3,4,5)‐triphosphate (PIP3) into phosphatidylinositol (4,5)‐bisphosphate (PIP2), thereby inactivating the PI3K‐AKT pathway.^13^, ^33^ These findings suggest an additional role for CPPED1 in regulation of the PI3K‐AKT pathway upstream of AKT1.

PAK4 is a member of the p21‐activated kinase family and a serine/threonine kinase. PAK4 acts as a signalling integrator to regulate different cellular processes, including cell motility, metabolism, cell cycle, proliferation, transformation, inflammation and gene expression.^34^, ^35^ In addition to its various cellular functions, PAK4 has an important role in different cancers via activation of pathways such as the PI3K‐AKT signalling pathway.^36^, ^37^, ^38^, ^39^, ^40^, ^41^, ^42^ In particular, PAK4 is required for maximal phosphorylation of AKT1.^22^ PRP4 kinase is important for regulation cell growth and survival of cancer, and it is reported to phosphorylate Ser104 on PAK4. How phospho‐Ser104 affects PAK4 function is unknown.^43^ Here, we show that CPPED1 dephosphorylates Ser104. However, whether the dephosphorylation of Ser104 on PAK4 by CPPED1 affects the PI3K–AKT signalling pathway remains to be elucidated.

**FIGURE 5:**
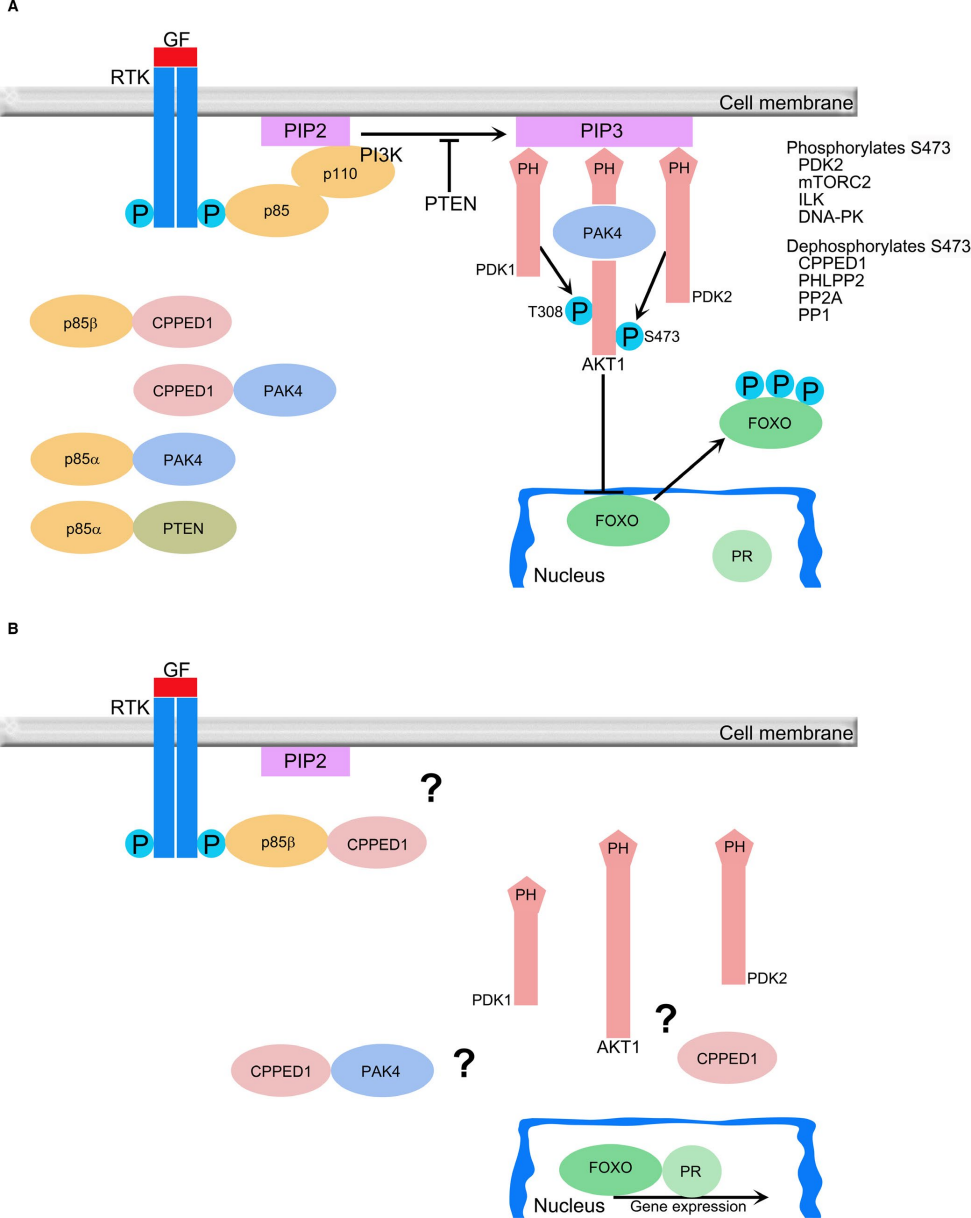
CPPED1 involvement with PI3K‐AKT pathway. Schematic view of activated (A) and inactivated (B) PI3K‐AKT pathway. PI3K‐AKT is a conserved pathway that is involved in many types of cellular processes. When growth factor (GF) binds to receptor tyrosine kinase (RTK), RTK tyrosine residues are phosphorylated. Regulatory subunit p85 binds to phosphorylated tyrosine and brings catalytic subunit p110 adjacent to substrate PIP2. Once PIP3 is formed from PIP2 by PI3K, pleckstrin homology (PH) domains of PDK1, PDK2 and AKT1 bind to PIP3, which results in phosphorylation and activation of AKT1.^30^ PDK1 phosphorylates Thr‐308. Ser‐473 is phosphorylated by various proteins such as PDK2, integrin‐linked kinase (ILK), mechanistic target of rapamycin complex (mTORC2) and DNA‐dependent protein kinase (DNA‐PK).^52^, ^53^ PAK4 has been shown to bind to p85α (also known as PIK3R1) and is required for maximal phosphorylation of AKT1.^22^ By contrast, protein phosphatase 2A (PP2A),^54^ protein phosphatase 1 (PP1),^55^ PH domain leucine‐rich repeat protein phosphatase 2 (PHLPP2)^56^ and CPPED1^11^ are known to dephosphorylate Ser‐473 and inactivate AKT1. Among other functions, activated AKT1 phosphorylates transcription factor FOXO1, which results in nuclear exclusion of phosphorylated FOXO1.^57^ This might also prevent formation of the FOXO‐progesterone receptor (PR) transcription factor complex.^48^, ^49^, ^50^ By contrast, PI3K‐AKT pathway inhibition allows for formation of the FOXO‐PR complex. We found that CPPED1 bound p85β (also known as PIK3R2) and PAK4, which could have an effect on the PI3K‐AKT Pathway. We also found that CPPED1 mediated the effect of progesterone on the expression of a set of genes.

PIK3R2, the other characterized binding partner of CPPED1, is a regulatory subunit of PI3K.^44^ There are different classes of PI3Ks. Class I PI3Ks comprise p110 catalytic and p85 regulatory subunits (Figure 5A). There are different forms of p110 and p85 subunits, such as p110α or p110β and p85α (PIK3R1) and p85β (PIK3R2).^45^ p110α and p110β regulate cell cycle entry,^46^ and regulatory subunits PIK3R1 and PIK3R2 stabilize these catalytic subunits.^45^ When bound to the catalytic subunits, the regulatory subunits inhibit the activity of PI3K. Upon activation of receptor tyrosine kinase (RTK), the regulatory subunits bind to RTK and liberate PI3K, which results in PI3K activation and localization of PI3K adjacent to its substrate PIP2 at the cell membrane ^30^, ^45^ (Figure 5A). One other way to activate PI3K is via RAS in which RAS activates p110 catalytic subunit independently of p85.^47^

In the current study, we found that the CPPED1‐PIK3R2 complex was located on the cytosolic side of the cell membrane. Formation of the CPPED1‐PIK3R2 complex could affect localization of PI3K at the cell membrane, thereby inactivating the PI3K‐AKT pathway (Figure 5B), or the complex could bring CPPED1 to an as‐yet unidentified target on the cell membrane. In addition, we identified a CPPED1‐PAK4 complex. This complex could also affect activity of the PI3K‐AKT pathway, because PAK4 is required for maximal phosphorylation of AKT1.^22^

If CPPED1 inhibits the PI3K‐AKT pathway by binding to PIK3R2 or PAK4, or by dephosphorylating AKT1, this could enable nuclear localization of FOXO1, formation of the FOXO1‐PR transcription complex, and expression of genes that maintain pregnancy (Figure 5B). Namely, FOXO1 and progesterone receptor (PR) are located in the same transcriptional complex, which results in the activation of genes coding for senescence‐associated cell cycle inhibitors and decidualization.^48^, ^49^ Progesterone binds to PR and maintains pregnancy.^50^ In addition, androgen, oestrogen and follicle‐stimulating hormone receptors are FOXO1 transcription factor‐binding partners.^51^ During spontaneous term labour, the amount of CPPED1 decreases.^13^ This could result in the activation of the PI3K‐AKT pathway (Figure 5A), nuclear exclusion of FOXO1, disassembly of the FOXO1‐PR complex, and initiation of labour. When we treated HTR8/SVneo cells with progesterone, we found that expression levels of certain genes either increased or decreased. By simultaneously silencing *CPPED1* expression and treating with progesterone, we identified a subset of genes that no longer responded to progesterone treatment. These findings suggest that CPPED1 is required to mediate the effect of progesterone on the expression of certain genes in human placental HTR8/SVneo trophoblast cells.

A growing body of evidence indicates that CPPED1 is an important regulator of the PI3K‐AKT signalling pathway. In the placenta, CPPED1 levels are associated with the timing of birth. However, the exact role of CPPED1 in placental trophoblast cells is still unclear. The exact downstream effects of decreased *CPPED1* expression during spontaneous labour remain to be established but the changes could be mediated via the PI3K‐AKT‐FOXO/PR axis.

## CONFLICT OF INTEREST

The authors confirm that there are no conflicts of interest.

## AUTHOR CONTRIBUTION

**Antti M Haapalainen:** Data curation (equal); Formal analysis (equal); Funding acquisition (equal); Investigation (equal); Methodology (equal); Supervision (equal); Validation (equal); Visualization (equal); Writing‐original draft (equal); Writing‐review & editing (equal). **Ravindra Daddali:** Data curation (equal); Formal analysis (equal); Investigation (equal); Methodology (equal); Validation (equal); Visualization (equal); Writing‐original draft (equal); Writing‐review & editing (equal). **Mikko Hallman:** Funding acquisition (equal); Investigation (equal); Methodology (equal); Project administration (equal); Resources (equal); Supervision (equal); Validation (equal); Writing‐original draft (equal); Writing‐review & editing (equal). **Mika Rämet:** Funding acquisition (equal); Investigation (equal); Methodology (equal); Project administration (equal); Resources (equal); Supervision (equal); Validation (equal); Writing‐original draft (equal); Writing‐review & editing (equal).

## Supporting information

Supplementary Material

## Data Availability

Transcriptomic data were deposited into the National Center for Biotechnology Information's Gene Expression Omnibus and are accessible through GEO Series accession number GSE149640 (https://www.ncbi.nlm.nih.gov/geo/query/acc.cgi?acc=GSE149640).
