# Supplementary material for: Human CPPED1 belongs to calcineurin‐like metallophosphoesterase superfamily and dephosphorylates PI3K‐AKT pathway component PAK4
Source: J Cell Mol Med. 2021 May 19;25(13):6304–17. doi: 10.1111/jcmm.16607 (PMC8366450; doi:10.1111/jcmm.16607)
Supplement: Supplementary file 1 — Supplementary Material [file JCMM-25-6304-s001.docx]

**SUPPORTING INFORMATION**

**Content:** Materials and Methods (extended), Tables S1–S4, Figures S1–S13

**MATERIALS AND METHODS (extended)**

**Construction of plasmids for protein microarray, coimmunoprecipitation, and bimolecular fluorescence complementation experiments**

Prior to the protein microarray experiment, CPPED1 was cloned into the PSF-OXB20-NH2-6HIS-V5-TEV dual-tag plasmid (Sigma-Aldrich). For CoIP, CPPED1 was cloned into the pCMV-Flag 2B plasmid and both PAK4 and PIK3R2 were cloned into pcDNA3-myc plasmids (Invitrogen). To visualize protein–protein interactions by BiFC, CPPED1 was cloned into pBiFC-VN173 plasmid (CPPED1-VN) and PAK4 and PIK3R2 were cloned into pBiFC-VC155 plasmids (PAK4-VC and PIK3R2-VC, respectively). pBiFC-VN173 (Addgene plasmid #22010) and pBiFC-VC155 (Addgene plasmid #22011) were gifts from Chang-Deng Hu [16]. All genes were of human origin. Final clones were confirmed by restriction enzyme double digestion, and the coding frame was confirmed by sequencing. Primers used for cloning into different plasmids are listed in Table S1.

**Recombinant CPPED1 expression and purification**

*E. coli* BL21(DE3) cells were lysed in Ni-NTA A-buffer (50 mM HEPES, 0.5 M NaCl, and 20 mM imidazole; pH 8.0) with 10 mM phenylmethylsulfonyl fluoride, and the protein lysate was loaded onto a Ni-NTA column that had been equilibrated with Ni-NTA A-buffer. Bound proteins were eluted with Ni-NTA B-buffer (50 mM HEPES, 0.5 M NaCl, and 0.5 M imidazole; pH 8.0). Eluted fractions that contained CPPED1 were pooled and dialyzed overnight against HIC A-buffer (8 mM Na_2_HPO_4_, 2 mM KH_2_PO4, 2.7 mM KCl, 137 mM NaCl, and 1.5 M (NH_4_)_2_SO_4_; pH 7.4). Overnight-dialyzed sample was loaded onto a HIC column (1ml) that had been equilibrated with HIC A-buffer. The column was eluted with HIC B-buffer (8 mM Na_2_HPO_4_, 2 mM KH_2_PO4, 2.7 mM KCl, and 137 mM NaCl; pH 7.4) with a 20 ml linear gradient from 0 to 100% HIC B-buffer. Fractions (1 ml) that contained CPPED1 were pooled and applied to a size exclusion chromatography column that had been equilibrated with HEPES buffer (20 mM HEPES and 150 mM NaCl, pH 8.0). Fractions (1 ml) were collected at a flow rate of 0.75 ml/min. Fractions that contained human recombinant CPPED1 were combined. Purified CPPED1 was concentrated to 0.5 mg/ml, aliquoted into 50 µl fractions, immediately snap-frozen in liquid nitrogen, and stored at −80°C until it was used in experiments. CPPED1 purity was analyzed by SDS-PAGE (Fig. S1) with 12% Bis-Tris gel (NuPAGE Novex, Life Technologies) in accordance with the manufacturer’s instructions.

**Identification of recombinant CPPED1 by western blot**

First, the CPPED1 protein sample was separated by SDS gel electrophoresis and electrotransferred onto a 0.45 μm nitrocellulose membrane (Thermo Scientific), as described previously [13]. Membranes were incubated in blocking solution (Odyssey Blocking Buffer [TBS], LI-COR Biosciences) at room temperature for 1 h. To detect CPPED1 and V5, we used rabbit anti-human CPPED1 antibody (HPA040938, 1:250 dilution; Sigma-Aldrich) and mouse anti-V5 antibody (V8012, 1:2000 dilution; Sigma-Aldrich), respectively. The primary antibody incubation was done overnight at 4°C. Secondary antibodies were goat anti-rabbit IgG DyLight 680 conjugate (611-144-002-0.5, 1:10,000 dilution; Rockland) and goat anti-mouse IgG DyLight 800 conjugate (610-145-002-0.5, 1:10,000 dilution; Rockland). Secondary antibody incubations were done at room temperature for 1 h. Detection was performed with the Odyssey Infrared Imaging System (LI-COR Biosciences).

**Detection of CPPED1 phosphatase activity by western blot**

In the phosphatase assay, phosphorylation levels of Ser473 (pS473) of AKT1 were determined with pS473-specific antibody [13], and the amount of pS473 was compared with the total amount of AKT1 (Fig. 1B). To quantify phosphorylation, we used rabbit anti-human AKT1 pS473 antibody (9018S, 1:1000 dilution; Cell Signaling Technology) and mouse anti-human AKT1 antibody (2967S, 1:1000 dilution; Cell Signaling Technology). Secondary antibodies were goat anti-rabbit IgG Dylight 680 conjugate (611-144-002-0.5, 1:10,000 dilution; Rockland) and goat anti-mouse IgG Dylight 800 conjugate (610-145-002-0.5, 1:10,000 dilution; Rockland). Detection was done with the Odyssey Infrared Imaging System (LI-COR Biosciences) (Fig. S13). We used 87 ng of human recombinant AKT1 (009-001-P21; Rockland) as a run calibrator for western blots.

**Circular dichroism and static light scattering**

CD measurements were acquired every 1 nm, with 0.5 s as integration time, and repeated three times with baseline correction. Data were processed with Chirascan Pro-Data Viewer (Applied Photophysics) and CDNN (<http://www.xn--gerald-bhm-lcb.de/download/cdnn>). Direct CD measurements (θ; mdeg) were converted into mean residue molar ellipticity ([θ]_MR_) by Pro-Data Viewer. In total, we analyzed 8 µg of recombinant CPPED1 in PBS (8 mM Na_2_HPO_4_, 2 mM KH_2_PO4, 2.7 mM KCl, and 137 mM NaCl; pH 7.4).

ASTRA (Wyatt Technology) was used in mass determination. Sample and column were equilibrated with the same buffer (20 mM HEPES and 0.15M NaCl, pH 8.0). Aliquots of 13 µM protein sample (10 µl) were used for each injection.

**Human proteome microarray**

Prior to incubation with bait protein (human recombinant V5-tagged CPPED1), the human proteome microarray was blocked with blocking buffer (2 % BSA, 20 mM HEPES, and 150 mM NaCl; pH 8.0) at 4°C overnight. V5-tagged CPPED1 was diluted to a final concentration of 40 μg/ml in blocking buffer and added to the microarray once the blocking buffer had been removed from the microarray. V5-tagged CPPED1 was incubated with the microarray at room temperature for 2 h with gentle shaking. The array was washed three times with HEPES buffer (20 mM HEPES and 150 mM NaCl, pH 8.0), followed by two 5 min washes with HEPES buffer. The washing solution was always completely removed. Fluorophore conjugated anti-V5-649 antibody (600-443-378, 1:1000 in blocking buffer; Rockland) and anti-GST-550 antibody (0.65 μg/ml in blocking buffer) were then incubated for 2 h at room temperature on the HuProt™ array with gentle shaking. The array was washed three times with HEPES buffer, followed by two 5 min washes with HEPES buffer, then rinsed with water, and finally dried by spindown. Upon completion of the assay, the arrays were scanned on a Tecan LS400 microarray scanner at 532 nm (to detect GST staining of all proteins) and 633 nm (to detect interactions with CPPED1) excitation, with a resolution of 10 μm. Fluorescence data were collected and fluorescence intensity measurements for each protein on the negative control array were subtracted to yield protein-specific fluorescence signals (Table 1). Positive and negative control assays were performed in parallel with the sample. In the negative control assay, one additional microarray was treated identically to the experimental array except CPPED1 was omitted. This allowed us to eliminate proteins that exhibited significant signal due to interactions with the secondary (anti-V5) detection reagents from the analysis. For the positive control, the microarrays were coincubated with 0.65 μg/ml goat polyclonal anti-GST antibody conjugated to a fluorophore with excitation at 550 nm (anti-GST-550). This provided data on the relative amounts of all proteins on the array.

**Fluorescence colocalization analysis**

To detect CPPED1, AKT1, PAK4, and PIK3R2 we used rabbit anti-human CPPED1 antibody (HPA040938, 1:500 dilution; Sigma-Aldrich), mouse anti-human AKT1 antibody (sc-5298, 1:500 dilution, Santa Cruz), mouse anti-human PAK4 antibody (sc-390507, 1:250 dilution; Santa Cruz), and mouse anti-human PIK3R2 antibody (sc-515646, 1:500 dilution; Santa Cruz), respectively. Primary antibody incubations were done at room temperature for 1 hour. Secondary antibodies were goat anti-rabbit IgG Alexa Fluor 488 conjugate (S4412, 1:500 dilution; Cell Signaling Technology) and goat anti-mouse IgG Alexa Fluor 594 conjugate (S8890, 1:500 dilution; Cell Signaling Technology). Cells and tissue samples were observed with Leica SP8 FALCON laser scanning confocal microscope and images were acquired wit LAS X software. Excitation wavelengths were 509 nm for Alexa Fluor 488 conjugate and 608 nm for Alexa Fluor 594 conjugate. Emission wavelength were 593 nm and 782 nm. Objective used was HC PL APO 63×/1.40 OIL CS2 DIC (oil). Primary antibodies were omitted in the negative controls.

**Bimolecular fluorescence complementation assay**

After 18 h of transfection, cells were washed once with PBS (pH 7.4), fixed with 4% formaldehyde solution in PBS (pH 7.4) for 20 min at room temperature, and washed three times with PBS (pH 7.4). After fixation, cells were observed with an Olympus FluoView FV1000 confocal microscope and images were acquired. Excitation wavelength was 488 nm, and emission wavelength was 510 nm. Objectives used were UPLSAPO 20X/0.75 or UPLSAPO 60X/1.35 oil.

**Coimmunoprecipitation**

HEK293T cells were seeded in 60 mm dishes and grown overnight in DMEM (Invitrogen Life Technologies) supplemented with 10% fetal bovine serum (Invitrogen Life Technologies), 100 U/ml penicillin, and 100 mg/ml streptomycin. Plasmid DNA–Lipofectamine 3000 (Invitrogen Life Technologies) complexes were prepared in serum-free medium.

To detect CPPED1, flag- tags, myc- tags, PAK4, and PIK3R2, we used rabbit anti-human CPPED1 antibody (HPA040938, 1:250 dilution; Sigma-Aldrich), rabbit anti-flag antibody (F7425, 1:400 dilution; Merck), mouse anti-myc antibody (sc-40, 1:200 dilution; Santa Cruz), mouse anti-human PAK4 antibody (sc-390507, 1:100 dilution; Santa Cruz), and mouse anti-human PIK3R2 antibody (sc-515646, 1:100 dilution; Santa Cruz), respectively. Primary antibody incubations were done overnight at 4°C. Secondary antibodies were goat anti-rabbit IgG DyLight 680 conjugate (611-144-002-0.5, 1:10,000 dilution; Rockland) and goat anti-mouse IgG DyLight 800 conjugate (610-145-002-0.5, 1:10,000 dilution; Rockland). Secondary antibody incubations were done at room temperature for 1 h. Detection was performed with the Odyssey Infrared Imaging System (LI-COR Biosciences).

**Phosphatase assay detection by mass spectrometry**

After the phosphatase assay, the samples were treated as follows: a 50 µl protein solution was treated with 2.5 µl of 1 M DTT in water and incubated for 30 min at 37°C, after which 5 µl of 1 M iodoacetamide (in 50% methanol) was added and the solution was incubated for another 30 min. The pH was checked, and if it was in the acidic range, 3 µl of 1 M ammonium bicarbonate was added before acetone was added to 80% (v/v) and the solution was incubated overnight at −20°C. After centrifugation for 30 min at 15,000 × *g* and 4°C, the pellet was washed with 200 µl of cold 80% (v/v) acetone/water, stored for 2 h at 20°C, centrifuged as before, dried briefly in a speedvac, and digested with trypsin (Sigma proteomics grade T7575, 2 ng/µl in 40 mM ammonium bicarbonate containing 9% acetonitrile; 10 ul volume) overnight at 37 °C.

Trifluoroacetic acid was added to 0.5% (v/v), and the sample was vortexed and sonicated for 20 min in an ultrasonic bath and clarified by centrifugation as above. A 4 µl sample of the supernatant was subjected to LC/MS on an Easy Nano LC 1000 (Thermo) linked to an Orbitrap Lumos mass spectrometer (Thermo). The chromatography system comprised a Symmetry C18 0.18 × 20 mm trap column and a peptide BEHC18 (1.7 µm particles) 0.075 × 150 mm analytical column (both Waters). Chromatography took place with 0.3 µl/min flow and a linear gradient from 3% to 35% over 90 min (0.1% formic acid in water to 0.1% FA in acetonitrile). Between sample runs, the column was washed four times with a dedicated method featuring a sawtooth gradient going from 20% up to 80% acetonitrile.

The mass spectrometer was operated in data-dependent acquisition mode with 3 s cycles, where the first 100 ms was dedicated to MS scans m/z 350 to 1500, automatic gain control (AGC) 4e5 at resolution 120,000. The remaining cycle time was used for MS/MS interrogation with higher-energy collisional dissociation fragmentation at 30% relative collision energy. Ion trap (IT) and orbitrap (OT) mass analyzers were used in parallel, where a threshold of 2.5e4 was set for orbitrap acquisition, while ion counts between 1e3 and 2.5e4 were passed to the ion trap. AGC targets were set to 1e4 and 5e4 for IT and OT, respectively, and maximum injection time was set to 200 ms (OT, resolution 15,000) and 300 ms (IT) with the “AAPT” option activated (we later discovered that this option was compromised by a software flaw and did not operate properly).

Raw data were processed with “Proteom Discoverer” (Thermo), and we used the Sequest algorithm to search the Swissprot-human database (v 2017-07-05). Parameters included tryptic digestion with up to two missed cleavages, Cys compulsorily modified with carbamidomethyl, optional Met oxidation, Gln and Asn deamidation, Ser/Thr/Tyr phosphorylation, and protein N-terminus acetylation. Identification was cut off at 1% false discovery rate as determined with a decoy database (Percolator), and label-free quantification based on precursor ion area normalized to total peptides was employed.

**Transfection of small interfering RNAs and progesterone treatment of transfected HTR8/SVneo cells**

In the first experiment, after 24 h of second transfection, siNEG cells were treated with 100 ng of progesterone (P4) that had been dissolved in 0.1% ethanol. Transcriptomes of these cells were then compared with the transcriptomes of the siNEG cells treated only with 0.1% ethanol to obtain a list of genes in which expression levels were affected by P4 (Tables S2, S3). P4 and ethanol incubation times were 24 h. In the second experiment, siCPPED1 cells were treated with 100 ng of P4; transcriptomes of these cells were compared with the gene list from the first experiment to yield a list of genes in which expression levels were affected by simultaneous silencing of *CPPED1* expression and P4 treatment (Table S4). All samples were measured in triplicate. Significant differences in expression levels were identified by nonparametric Kruskal–Wallis test, in which significance values were adjusted by the Bonferroni correction for multiple ties.

**Table S1.** Primers used to clone *CPPED1,* *PAK4,* and *PIK3R2* into different plasmids**.** (F), forward and (R), reverse primers. Restriction enzyme sites underlined, and corresponding restriction enzyme named at end of primer.

| **Name of plasmid** | **Primers used in PCR to obtain insert for ligation** |
| --- | --- |
| CPPED1- PSF-OXB20 | **(F)** GCAT GAATTC G ATG TCG GCT GCA GAG GCG GG (*Eco*R1)  **(R)** GC GGATCCTCA TTT TTT CTT GAT CAA ATC CAT GAG ATC (*Bam*H1) |
| CPPED1-pCMV-Flag 2B | **(F)** GC GAATTC G ATG TCG GCT GCA GAG GCG GG (*Eco*R1)  **(R)** GC GGATCC TTT TTT CTT GAT CAA ATC CAT GAG ATC (*Bam*H1) |
| CPPED1-pBiFC-VN173 | **(F)** GAGC AAGCTT ATG TCG GCT GCA GAG GCG GGG (*Hind*III)  **(R)** GACC GTCGAC TTT TTT CTT GAT CAA ATC CAT GAG ATC (*Sal*1) |
| PAK4-pcDNA3-myc | **(F)** GGCC AAGCTT ATGTTTGGGAAGAGGAAGAAGCGG (*Hind*III)  **(R)** GGCC CTCGAG TCTGGTGCGGTTCTGGCGCATG (*Xho*1) |
| PAK4-pBIFC-VC155 | **(F)** GAGC Gaattc tt ATGTTTGGGAAGAGGAAGAAGCGG (*Eco*R1)  **(R)** GACG ggt acc TCTGGTGCGGTTCTGGCGCATG (*Kpn*1) |
| PIK3R2- pcDNA3-myc | **(F)** GGCC GAATTC ATGGCGGGCCCTGAGGGCTTCC (*Eco*R1)  **(R)** GGCC CTCGAG GCGGGCGGCAGGCGGCGGGCC (*Xho*1) |
| PIK3R2-pBIFC-VC155 | **(F)**GAGC Gaattc tt ATGGCGGGCCCTGAGGGCTTCC (*Eco*R1)  **(R)** GGAC CTCGAG C GCGGGCGGCAGGCGGCGGGCC (*Xho*1) |

**Table S2. Downregulated genes after progesterone treatment in HTR8/SVneo cells.** The siNEG (sham siRNa) cells were treated with progesterone (P4) that had been dissolved in 0.1% ethanol. Transcriptomes of these cells were then compared with the transcriptomes of the siNEG cells treated only with 0.1% ethanol. Thresholds used in filtering the differentially expressed genes were: FC > 2.0 and FDR-adjusted *p*-value < 0.05. The differentially expressed genes have been ranked based both on *p*-value and fold change. For example, the first gene in the table (*MIR5581*) has the highest possible rank.

| **Gene name** | **FC^1^** | ***p* value^2^** | **adjusted *p* value^3^** | **EntrezID** | **Description** |
| --- | --- | --- | --- | --- | --- |
| *MIR5581* | -3.941 | 0.000031 | 0 | 100847010 | microRNA 5581 |
| *MIRLET7F1* | -3.941 | 0.000031 | 0 | 406888 | microRNA let-7f-1 |
| *TM4SF19* | -4.947 | 0.032746 | 0.010144 | 116211 | transmembrane 4 L six family member 19 |
| *SNORD2* | -3.398 | 0.033100 | 0.010337 | 619567 | small nucleolar RNA, C/D box 2 |
| *SLC15A1* | -2.473 | 0.030806 | 0.005735 | 6564 | solute carrier family 15 member 1 |
| *SNORA4* | -2.616 | 0.031498 | 0.007418 | 619568 | small nucleolar RNA, H/ACA box 4 |
| *KCTD16* | -2.644 | 0.032095 | 0.008668 | 57528 | potassium channel tetramerization domain containing 16 |
| *ZNF233* | -2.689 | 0.032298 | 0.008897 | 353355 | zinc finger protein 233 |
| *IL19* | -5.08 | 0.036266 | 0.016373 | 29949 | interleukin 19 |
| *SGCG* | -3.246 | 0.037606 | 0.018315 | 6445 | sarcoglycan gamma |
| *BCO1* | -2.367 | 0.035791 | 0.015497 | 53630 | beta-carotene oxygenase 1 |
| *C10orf131* | -2.453 | 0.036617 | 0.016903 | 100127889 | chromosome 10 open reading frame 131 |
| *TTPA* | -2.311 | 0.036235 | 0.016389 | 7274 | alpha tocopherol transfer protein |
| *WDR93* | -2.076 | 0.031883 | 0.008295 | 56964 | WD repeat domain 93 |
| *LOC101927604* | -3.372 | 0.041477 | 0.024865 | 101927604 | uncharacterized LOC101927604 |
| *LINC00235* | -3.913 | 0.042890 | 0.026936 | 64493 | long intergenic non-protein coding RNA 235 |
| *LRRC70* | -2.146 | 0.033498 | 0.010921 | 100130733 | leucine rich repeat containing 70 |
| *RNU6-45P* | -2.905 | 0.041830 | 0.025146 | 100873759 | RNA, U6 small nuclear 45, pseudogene |
| *WDR38* | -2.334 | 0.039864 | 0.022290 | 401551 | WD repeat domain 38 |
| *SNORD87* | -2.188 | 0.037029 | 0.017666 | 641648 | small nucleolar RNA, C/D box 87 |
| *TMEM5-AS1* | -2.845 | 0.043093 | 0.027050 | 104169670 | TMEM5 antisense RNA 1 |
| *ZG16B* | -2.858 | 0.043740 | 0.028661 | 124220 | zymogen granule protein 16B |
| *EID3* | -3.218 | 0.045705 | 0.031846 | 493861 | EP300 interacting inhibitor of differentiation 3 |
| *SNORA22* | -3.072 | 0.046411 | 0.032840 | 677807 | small nucleolar RNA, H/ACA box 22 |
| *MIR30E* | -3.072 | 0.046411 | 0.032911 | 407034 | microRNA 30e |
| *WFIKKN1* | -2.212 | 0.041017 | 0.024137 | 117166 | WAP, follistatin/kazal, immunoglobulin, kunitz and netrin domain containing 1 |
| *ATE1-AS1* | -2.227 | 0.042525 | 0.026614 | 100130887 | ATE1 antisense RNA 1 |
| *SNORD113-4* | -3.877 | 0.048467 | 0.036495 | 767564 | small nucleolar RNA, C/D box 113-4 |
| *MIR6772* | -3.899 | 0.049139 | 0.037049 | 102465463 | microRNA 6772 |
| *MIR503* | -3.093 | 0.048144 | 0.035945 | 574506 | microRNA 503 |
| *MIR4677* | -3.899 | 0.049139 | 0.037056 | 100616343 | microRNA 4677 |
| *MIR4648* | -3.093 | 0.048144 | 0.035949 | 100616116 | microRNA 4648 |
| *CD81-AS1* | -2.168 | 0.040447 | 0.022912 | 101927682 | CD81 antisense RNA 1 |
| *TBC1D29* | -2.372 | 0.045336 | 0.031431 | 26083 | TBC1 domain family member 29 |
| *C1orf234* | -4.987 | 0.051412 | 0.043043 | 729059 | chromosome 1 open reading frame 234 |
| *MIR450A2* | -3.919 | 0.051450 | 0.043115 | 574505 | microRNA 450a-2 |
| *FOCAD-AS1* | -3.919 | 0.051450 | 0.043119 | 101929548 | FOCAD antisense RNA 1 |
| *FDCSP* | -3.919 | 0.051450 | 0.043137 | 260436 | follicular dendritic cell secreted protein |
| *SNORA15* | -3.919 | 0.051450 | 0.043128 | 677803 | small nucleolar RNA, H/ACA box 15 |
| *SNORD50B* | -2.726 | 0.047920 | 0.035817 | 692088 | small nucleolar RNA, C/D box 50B |
| *MIR224* | -4.873 | 0.052564 | 0.044767 | 407009 | microRNA 224 |
| *MIRLET7A1* | -3.983 | 0.053348 | 0.045648 | 406881 | microRNA let-7a-1 |
| *USP12-AS2* | -3.557 | 0.052958 | 0.045431 | 100874071 | USP12 antisense RNA 2 (head to head) |
| *MIR1268A* | -4.004 | 0.054901 | 0.047772 | 100302233 | microRNA 1268a |
| *LOC102723780* | -2.584 | 0.051863 | 0.043282 | 102723780 | protein FRG1-like |
| *AGAP11* | -2.065 | 0.044150 | 0.029516 | 119385 | ArfGAP with GTPase domain, ankyrin repeat and PH domain 11 |

^1^Expression ratio (fold-change) between the compared sample groups. Comparison was between progesterone treated siNEG cells and siNEG cells.

^2^*t*-test *p* value for comparison between sample groups (progesterone treated siNEG cells and siNEG cells)

^3^FDR-adjusted *p* value

**Table S3. Upregulated genes after progesterone treatment in HTR8/SVneo cells.** The siNEG (sham siRNa) cells were treated with progesterone (P4) that had been dissolved in 0.1% ethanol. Transcriptomes of these cells were then compared with the transcriptomes of the siNEG cells treated only with 0.1% ethanol. Thresholds used in filtering the differentially expressed genes were: FC > 2.0 and FDR-adjusted *p*-value < 0.05. The differentially expressed genes have been ranked based both on *p*-value and fold change. For example, the first gene in the table (*HIST2H2AB*) has the highest possible rank.

| **Gene name** | **FC^1^** | ***p* value^2^** | **Adjusted**  ***p* value^3^** | **EntrezID** | **Description** |
| --- | --- | --- | --- | --- | --- |
| *HIST2H2AB* | 4.788 | 0.032422 | 0.009269 | 317772 | histone cluster 2 H2A family member b |
| *MIR3657* | 2.783 | 0.030949 | 0.005864 | 100500889 | microRNA 3657 |
| *MIR6785* | 2.787 | 0.031056 | 0.005986 | 102466911 | microRNA 6785 |
| *C1orf61* | 4.663 | 0.034596 | 0.013264 | 10485 | chromosome 1 open reading frame 61 |
| *LINC01023* | 2.843 | 0.032114 | 0.008665 | 100652853 | long intergenic non-protein coding RNA 1023 |
| *IL17C* | 4.862 | 0.035595 | 0.015048 | 27189 | interleukin 17C |
| *EPN2-IT1* | 2.278 | 0.030771 | 0.005470 | 100874309 | EPN2 intronic transcript 1 |
| *FFAR4* | 2.59 | 0.033088 | 0.010339 | 338557 | free fatty acid receptor 4 |
| *TEKT3* | 2.286 | 0.032202 | 0.008650 | 64518 | tektin 3 |
| *SNORD60* | 2.431 | 0.033708 | 0.011530 | 26788 | small nucleolar RNA, C/D box 60 |
| *PILRA* | 4.268 | 0.037736 | 0.018588 | 29992 | paired immunoglobin like type 2 receptor alpha |
| *REM2* | 3.798 | 0.037682 | 0.018357 | 161253 | RRAD and GEM like GTPase 2 |
| *CPSF4L* | 3.227 | 0.038002 | 0.018764 | 642843 | cleavage and polyadenylation specific factor 4 like |
| *MID1IP1-AS1* | 3.236 | 0.038464 | 0.019486 | 100874211 | MID1IP1 antisense RNA 1 |
| *LOC104968399* | 2.77 | 0.037164 | 0.017746 | 104968399 | uncharacterized LOC104968399 |
| *LINC00921* | 2.081 | 0.031755 | 0.008050 | 283876 | long intergenic non-protein coding RNA 921 |
| *RAB40AL* | 3.447 | 0.041406 | 0.024713 | 282808 | RAB40A, member RAS oncogene family-like |
| *ACY1* | 2.806 | 0.039635 | 0.021698 | 95 | aminoacylase 1 |
| *PYGM* | 2.507 | 0.039234 | 0.021161 | 5837 | phosphorylase, glycogen, muscle |
| *FAM221A* | 2.188 | 0.036835 | 0.017222 | 340277 | family with sequence similarity 221 member A |
| *CLEC4A* | 2.85 | 0.041908 | 0.025171 | 50856 | C-type lectin domain family 4 member A |
| *CRB3* | 2.274 | 0.039115 | 0.020838 | 92359 | crumbs 3, cell polarity complex component |
| *LOC101927056* | 2.057 | 0.035419 | 0.014849 | 101927056 | uncharacterized LOC101927056 |
| *PSPN* | 2.014 | 0.035757 | 0.015259 | 5623 | persephin |
| *NPIPB4* | 2.473 | 0.043372 | 0.027666 | 440345 | nuclear pore complex interacting protein family member B4 |
| *SAXO2* | 3.92 | 0.036885 | 0.017330 | 283726 | stabilizer of axonemal microtubules 2 |
| *LPAL2* | 2.406 | 0.044299 | 0.029461 | 80350 | lipoprotein(a) like 2, pseudogene |
| *TXNRD3NB* | 2.843 | 0.046075 | 0.032346 | 645840 | thioredoxin reductase 3 neighbor |
| *ASIC4* | 2.202 | 0.041375 | 0.024609 | 55515 | acid sensing ion channel subunit family member 4 |
| *LOC100129148* | 2.31 | 0.044180 | 0.029500 | 100129148 | uncharacterized LOC100129148 |
| *LINC00202-1* | 3.375 | 0.048618 | 0.036690 | 387644 | long intergenic non-protein coding RNA 202-1 |
| *SPATA4* | 2.36 | 0.045134 | 0.030958 | 132851 | spermatogenesis associated 4 |
| *SLAMF9* | 2.904 | 0.047165 | 0.034327 | 89886 | SLAM family member 9 |
| *NKX6-2* | 2.545 | 0.046433 | 0.032930 | 84504 | NK6 homeobox 2 |
| *FAM78B* | 2.501 | 0.046870 | 0.033771 | 149297 | family with sequence similarity 78 member B |
| *LUCAT1* | 2.446 | 0.047043 | 0.034157 | 100505994 | lung cancer associated transcript 1 (non-protein coding) |
| *FOXC2-AS1* | 2.192 | 0.043736 | 0.028664 | 103752587 | FOXC2 antisense RNA 1 |
| *ZNF793-AS1* | 2.738 | 0.049201 | 0.036998 | 101927720 | ZNF793 antisense RNA 1 (head to head) |
| *ABHD1* | 2.026 | 0.041645 | 0.025040 | 84696 | abhydrolase domain containing 1 |
| *SOAT2* | 2.545 | 0.049896 | 0.038012 | 8435 | sterol O-acyltransferase 2 |
| *TNFRSF6B* | 2.988 | 0.051874 | 0.043273 | 8771 | TNF receptor superfamily member 6b |
| *C11orf91* | 2.988 | 0.051874 | 0.043277 | 100131378 | chromosome 11 open reading frame 91 |
| *LGALS8-AS1* | 2.12 | 0.043006 | 0.027012 | 100287902 | LGALS8 antisense RNA 1 |
| *PPP1R32* | 2.15 | 0.043349 | 0.027678 | 220004 | protein phosphatase 1 regulatory subunit 32 |
| *SNORD96A* | 2.069 | 0.044517 | 0.030176 | 619571 | small nucleolar RNA, C/D box 96A |
| *SENP3-EIF4A1* | 2.147 | 0.045681 | 0.031857 | 100533955 | SENP3-EIF4A1 readthrough (NMD candidate) |
| *NDUFA4L2* | 2.028 | 0.045736 | 0.031826 | 56901 | NDUFA4, mitochondrial complex associated like 2 |
| *BCL2A1* | 2.303 | 0.055367 | 0.048798 | 597 | BCL2 related protein A1 |
| *PITRM1-AS1* | 2.263 | 0.054928 | 0.047857 | 100507034 | PITRM1 antisense RNA 1 |
| *HIST1H2BF* | 2.056 | 0.050530 | 0.038845 | 8343 | histone cluster 1 H2B family member f |
| *LINC00893* | 2.119 | 0.051816 | 0.043299 | 100131434 | long intergenic non-protein coding RNA 893 |
| *NANOS3* | 2.002 | 0.052218 | 0.044173 | 342977 | nanos C2HC-type zinc finger 3 |

^1^Expression ratio (fold-change) between the compared sample groups. Comparison was between progesterone treated siNEG cells and siNEG cells.

^2^*t*-test *p* value for comparison between sample groups (progesterone treated siNEG cells and siNEG cells)

^3^FDR-adjusted *p* value

**Table S4. The effect of simultaneous silencing of *CPPED1* expression and P4 treatment.** Each group (siNEG, siNEG+P4, and siCPPED1+P4) consisted of three replicates. For each replicate, normalized gene counts were calculated which were then used in calculating mean expression levels. The original list of genes are from Tables S2 and S3. Only those genes are shown that are affected by simultaneous silencing of *CPPED1* expression and P4 treatment. Light orange highlights those genes whose expression levels go up upon P4 treatment and returns to lover level after silencing of *CPPED1* expression (Fig. S12A). Similarly, light blue colour highlights those genes whose expression levels go down upon P4 treatment and returns to higher level after silencing of *CPPED1* expression (Fig. S12B). Genes whose expression levels change more than 50 % after silencing of *CPPED1* expression are bolded.

| **Gene name** | **Mean**  **siNEG^1^** | **Mean**  **siNEG+P4^2^** | **Mean**  **siCPPED1+P4^3^** |
| --- | --- | --- | --- |
| ***IL17C*** | 0.000 | 0.166 | 0.000 |
| *HIST2H2AB* | 0.000 | 0.379 | 0.288 |
| *C1orf61* | 0.033 | 0.342 | 0.198 |
| ***PILRA*** | 0.042 | 0.291 | 0.123 |
| *REM2* | 0.015 | 0.126 | 0.082 |
| ***RAB40AL*** | 0.028 | 0.208 | 0.000 |
| *LINC00202-1* | 0.031 | 0.136 | 0.072 |
| ***C11orf91*** | 0.061 | 0.287 | 0.025 |
| ***TNFRSF6B*** | 0.048 | 0.224 | 0.085 |
| ***SLAMF9*** | 0.049 | 0.222 | 0.036 |
| *TXNRD3NB* | 0.026 | 0.151 | 0.120 |
| ***MIR6785*** | 0.000 | 1.055 | 0.387 |
| *MIR3657* | 0.000 | 0.729 | 0.373 |
| *LOC104968399* | 0.101 | 0.343 | 0.290 |
| ***ZNF793-AS1*** | 0.063 | 0.268 | 0.086 |
| *SOAT2* | 0.160 | 0.444 | 0.296 |
| *PYGM* | 0.141 | 0.374 | 0.263 |
| ***NPIPB4*** | 0.055 | 0.154 | 0.072 |
| ***LUCAT1*** | 0.171 | 0.488 | 0.233 |
| ***SNORD60*** | 1.345 | 4.118 | 1.827 |
| *LPAL2* | 0.045 | 0.137 | 0.086 |
| *LOC100129148* | 0.303 | 0.843 | 0.440 |
| *BCL2A1* | 0.058 | 0.204 | 0.182 |
| *TEKT3* | 0.142 | 0.360 | 0.192 |
| *EPN2-IT1* | 0.102 | 0.310 | 0.262 |
| *CRB3* | 0.127 | 0.347 | 0.262 |
| *PITRM1-AS1* | 0.056 | 0.141 | 0.100 |
| *FAM221A* | 0.132 | 0.330 | 0.230 |
| *PPP1R32* | 0.291 | 0.664 | 0.581 |
| *SENP3-EIF4A1* | 0.059 | 0.140 | 0.083 |
| *LGALS8-AS1* | 0.135 | 0.326 | 0.258 |
| ***LINC00921*** | 0.069 | 0.167 | 0.076 |
| ***SNORD96A*** | 3.922 | 8.880 | 3.155 |
| *LOC101927056* | 0.159 | 0.367 | 0.205 |
| *NDUFA4L2* | 0.094 | 0.235 | 0.145 |
| *ABHD1* | 0.448 | 0.946 | 0.554 |
| *SAXO2* | 0.154 | 0.328 | 0.264 |
| *NANOS3* | 0.612 | 1.288 | 1.000 |
| *AGAP11* | 0.161 | 0.074 | 0.111 |
| *WDR93* | 0.256 | 0.120 | 0.162 |
| *LRRC70* | 0.586 | 0.268 | 0.348 |
| ***CD81-AS1*** | 0.142 | 0.055 | 0.127 |
| *SNORD87* | 7.736 | 3.367 | 6.054 |
| *WFIKKN1* | 0.378 | 0.164 | 0.310 |
| *ATE1-AS1* | 0.882 | 0.382 | 0.624 |
| *TTPA* | 0.258 | 0.106 | 0.208 |
| ***TBC1D29*** | 0.126 | 0.032 | 0.191 |
| ***C10orf131*** | 0.480 | 0.183 | 0.367 |
| *SLC15A1* | 0.198 | 0.075 | 0.111 |
| *LOC102723780* | 0.531 | 0.190 | 0.378 |
| ***SNORA4*** | 10.084 | 3.738 | 9.370 |
| ***KCTD16*** | 0.173 | 0.062 | 0.198 |
| ***TMEM5-AS1*** | 0.198 | 0.039 | 0.195 |
| ***RNU6-45P*** | 1.049 | 0.197 | 0.831 |
| ***SNORA22*** | 0.421 | 0.000 | 0.871 |
| ***MIR503*** | 0.805 | 0.000 | 1.079 |
| ***EID3*** | 0.134 | 0.029 | 0.088 |
| ***SGCG*** | 0.205 | 0.052 | 0.221 |
| ***LOC101927604*** | 0.353 | 0.080 | 0.352 |
| *SNORD2* | 10.354 | 2.766 | 4.766 |
| ***USP12-AS2*** | 0.284 | 0.054 | 0.121 |
| ***SNORD113-4*** | 1.113 | 0.000 | 0.285 |
| ***MIR6772*** | 1.297 | 0.000 | 0.329 |
| ***MIR4677*** | 0.519 | 0.000 | 0.263 |
| ***LINC00235*** | 0.227 | 0.039 | 0.131 |
| ***MIR5581*** | 1.406 | 0.000 | 0.728 |
| ***MIRLET7F1*** | 0.970 | 0.000 | 0.242 |
| ***MIR1268A*** | 0.306 | 0.000 | 0.230 |
| ***MIR224*** | 1.411 | 0.000 | 0.279 |
| ***C1orf234*** | 0.439 | 0.042 | 0.127 |
| ***IL19*** | 0.171 | 0.022 | 0.048 |

^1^Mean expression level for the specified sample group (siNEG HTR8/SVneo cells in the presence of 0.1 % ethanol)

^2^Mean expression level for the specified sample group (siNEG HTR8/SVneo cells in the presence of 100 ng of progesterone and 0.1 % ethanol)

^3^Mean expression level for the specified sample group (siCPPED1 HTR8/SVneo cells in the presence of 100 ng of progesterone and 0.1 % ethanol)

**FIGURES**

**
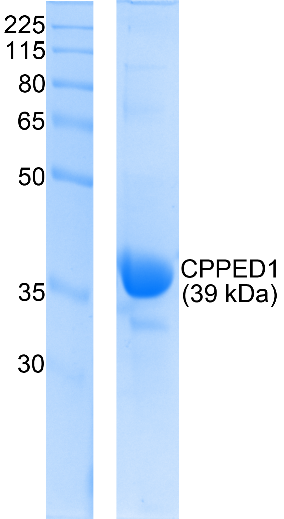
**

**Figure S1.** SDS-PAGE of purified human recombinant CPPED1. CPPED1 expressed as recombinant protein in *E. coli* and purified to apparent homogeneity with affinity, hydrophobic-interaction, and size-exclusion chromatography. Proteins separated on 12% Bis-Tris gel in NuPAGE MOPS SDS Running Buffer (Life Technologies). Gel is Coomassie stained. CPPED1 sample run on gel after Ni-NTA affinity column purification. Molecular weight markers in kilodaltons shown on left.

**
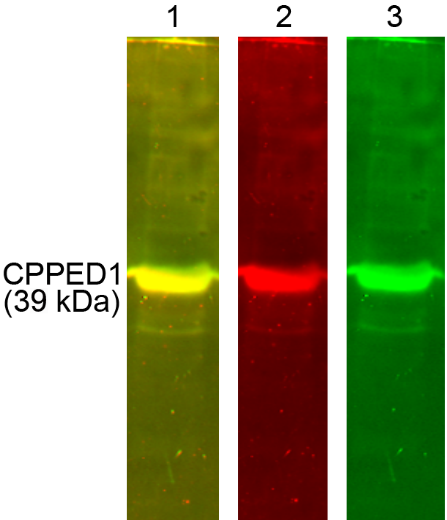
**

**Figure S2.** Densitometry evaluation of western blots of purified recombinant CPPED1. Recombinant CPPED1 expressed with N-terminal His-V5 dual tag. Lane 1, overlaid fluorescence signals of lanes 2 and 3; lane 2, human CPPED1 (red); and lane 3, V5 tag (green). Technical details are described in the Materials and Methods (Identification of recombinant CPPED1 by western blot).

**

**

**Figure S3.** Secondary structure analysis of CPPED1. Circular dichroism spectrum of human recombinant CPPED1. Spectra collected in the region of 200–280 nm.





**Figure S4.** Determination of CPPED1 monodispersity by static light scattering technique. Superdex 200 HR 5/150 GL (GE Healthcare) size-exclusion column and Wyatt miniDAWN TREOS static light scattering instrument (Wyatt Technology) were used. Plot of molecular mass and elution time shown, where peak is elution profile obtained by absorbance at 280 nm. Vertical dotted line represents light scattering signal shown as mass distribution.

**
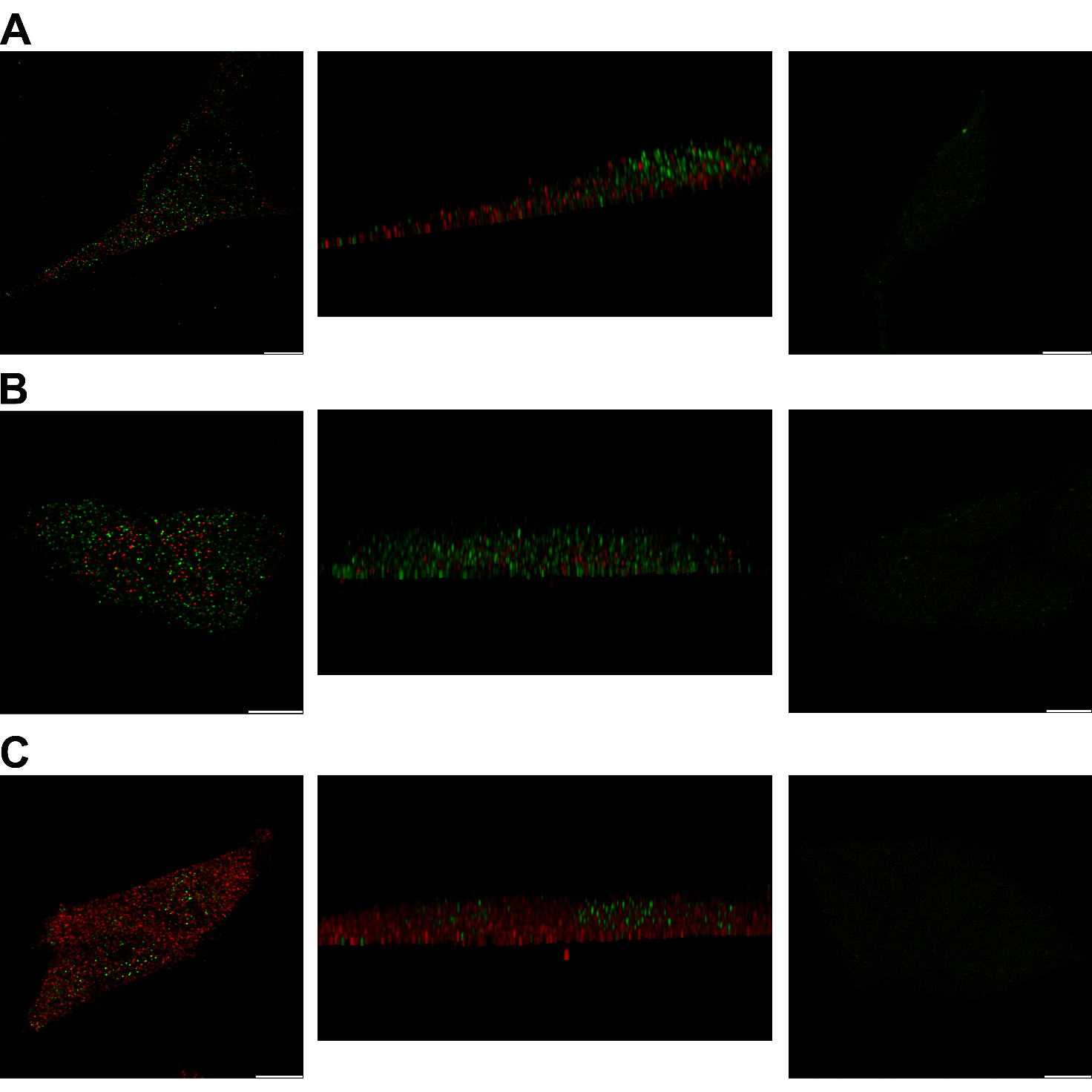
**

**Figure S5.** Confocal microscopy images of HTR8/SVneo cells expressing endogenous CPPED1, PAK4, PIK3R2, and AKT1. Colocalization fluorescence image of CPPED1 (green) and PAK4 (red) (A), CPPED1 (green) and (PIK3R2) (red) (B), and CPPED1 (green) and AKT1 (red) (C). Middle panel is a zoomed-in side view of the left panel. Panels on the right show negative controls treated the same way as samples but with primary antibody omitted. Objective used was HC PL APO 63×/1.40 OIL CS2 DIC (oil). Scale bar represents 10 µm.

**
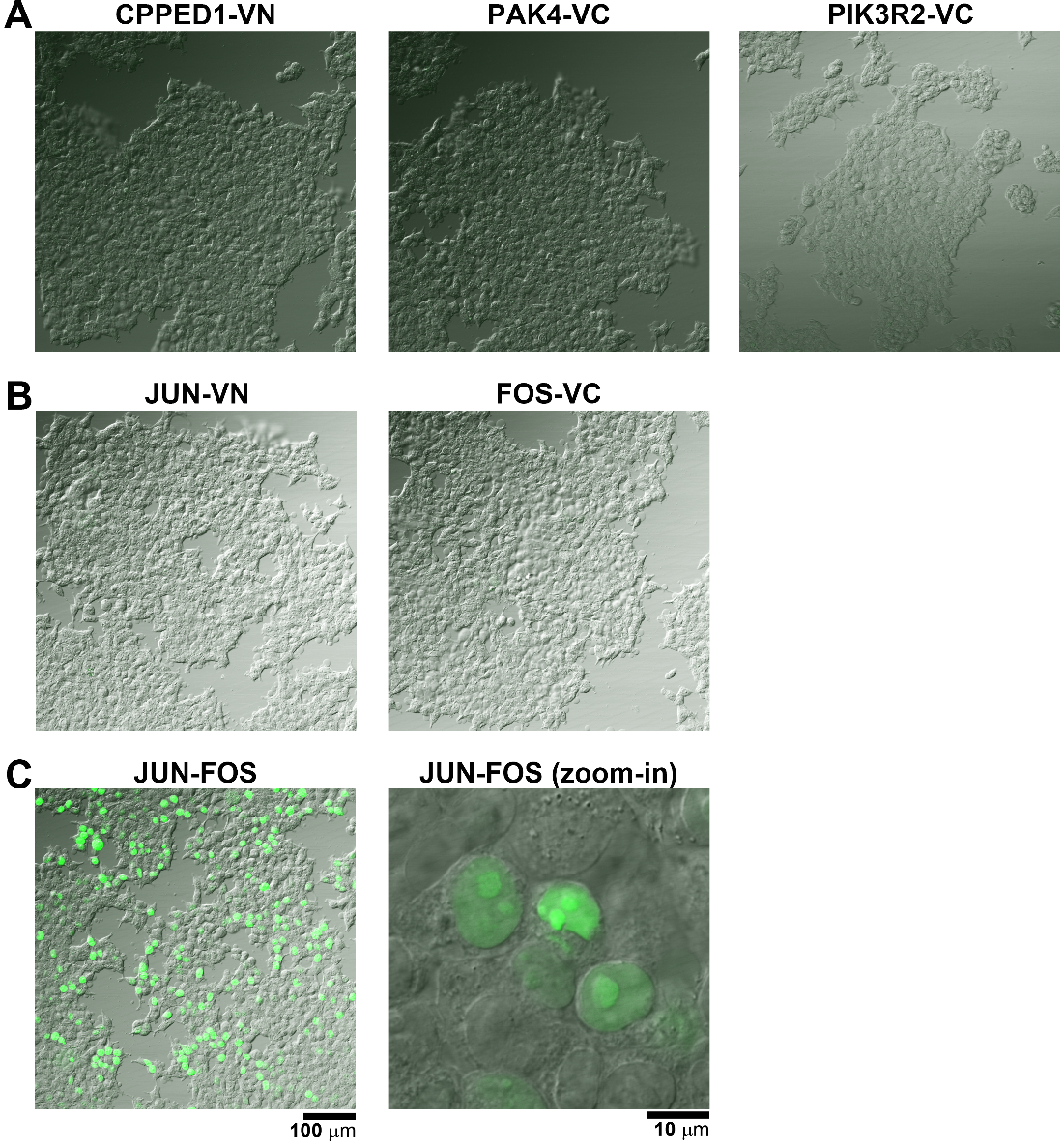
**

**Figure S6.** Fluorescence microscopy images of controls for bimolecular fluorescence complementation experiment. Confocal fluorescence images of HEK293T cells expressing CPPED1-VN, PAK-VC, or PIK3R2-VC (A); JUN-VN or FOS-VC (B); and JUN-VN and FOS-VC (C). CPPED1 and JUN expressed as fusion proteins with N-terminal fragment of Venus yellow fluorescence protein (YFP). PAK4, PIK3R2, and FOS expressed as fusion proteins with C-terminal fragment of Venus YFP. (A) and (B) are controls to confirm that fusion proteins expressed individually did not show fluorescence signal. (C) Cells that expressed both JUN-VN and FOS-VC had intact Venus YFP and consequent fluorescence signal. Cells transfected with 125 ng of each plasmid (A, B). Objectives: UPLSAPO 20X/0.75 (A, B, and C) and UPLSAPO 60X/1.35 oil (C, zoomed-in image). Scaled bar (100 µm) same for all images except zoomed-in image.

**
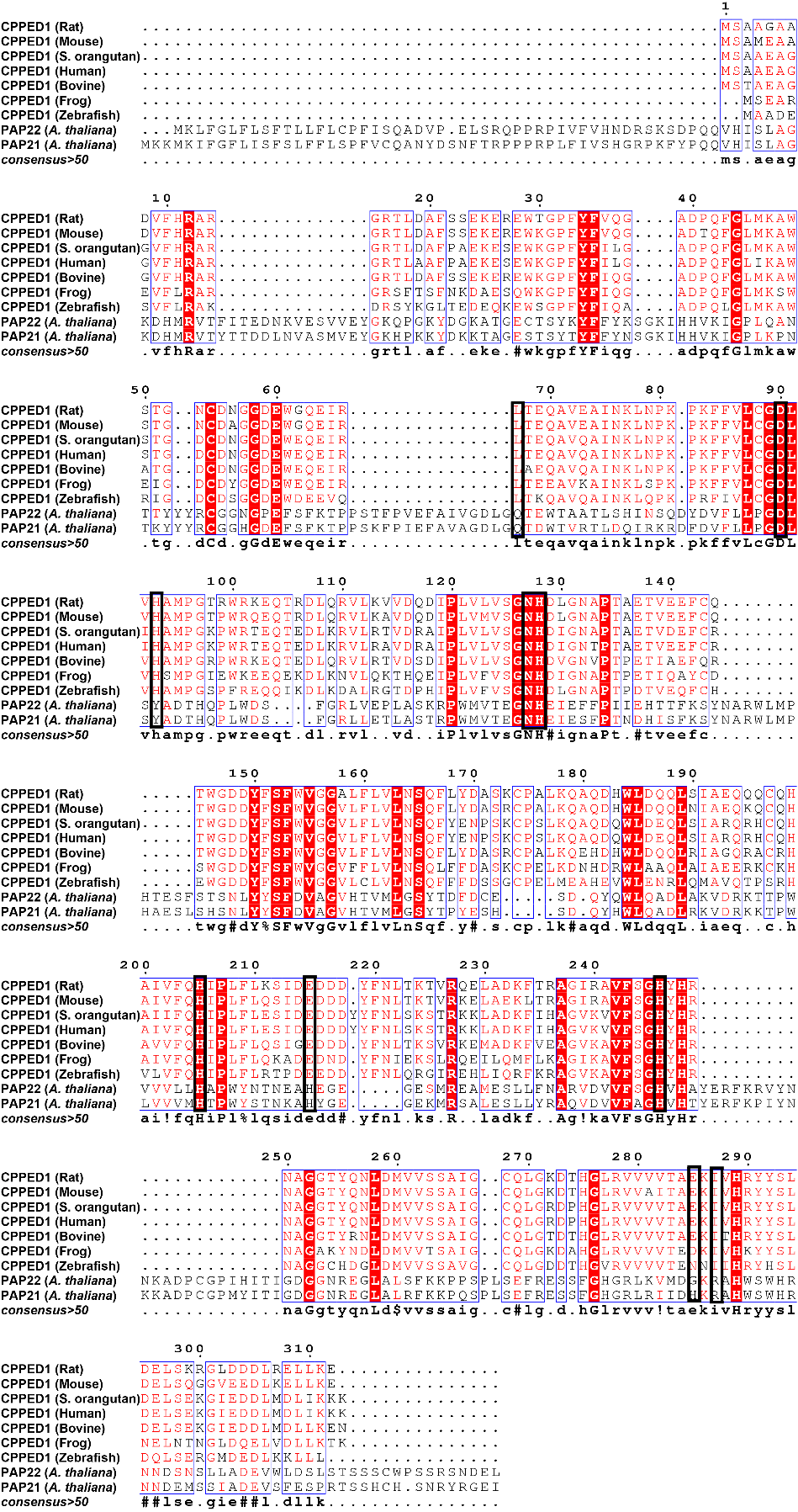
**

**Figure S7.** Alignment of CPPED1 with purple acid phosphatases. CPPED1 homologous sequences were searched with NPS@ software [14]. CPPED1 showed similarities with purple acid phosphatases (PAPs). Seven eukaryotic CPPED1 sequences were aligned with the two closest PAP homologs from *Arabidopsis thaliana* (*A. thaliana*). Amino acid residues conserved throughout the sequences highlighted in red. Similar residues denoted by red letters. Framed residues are important for activity, according to what is known of PAPs. Figure prepared with ESPript 3.0 [15].


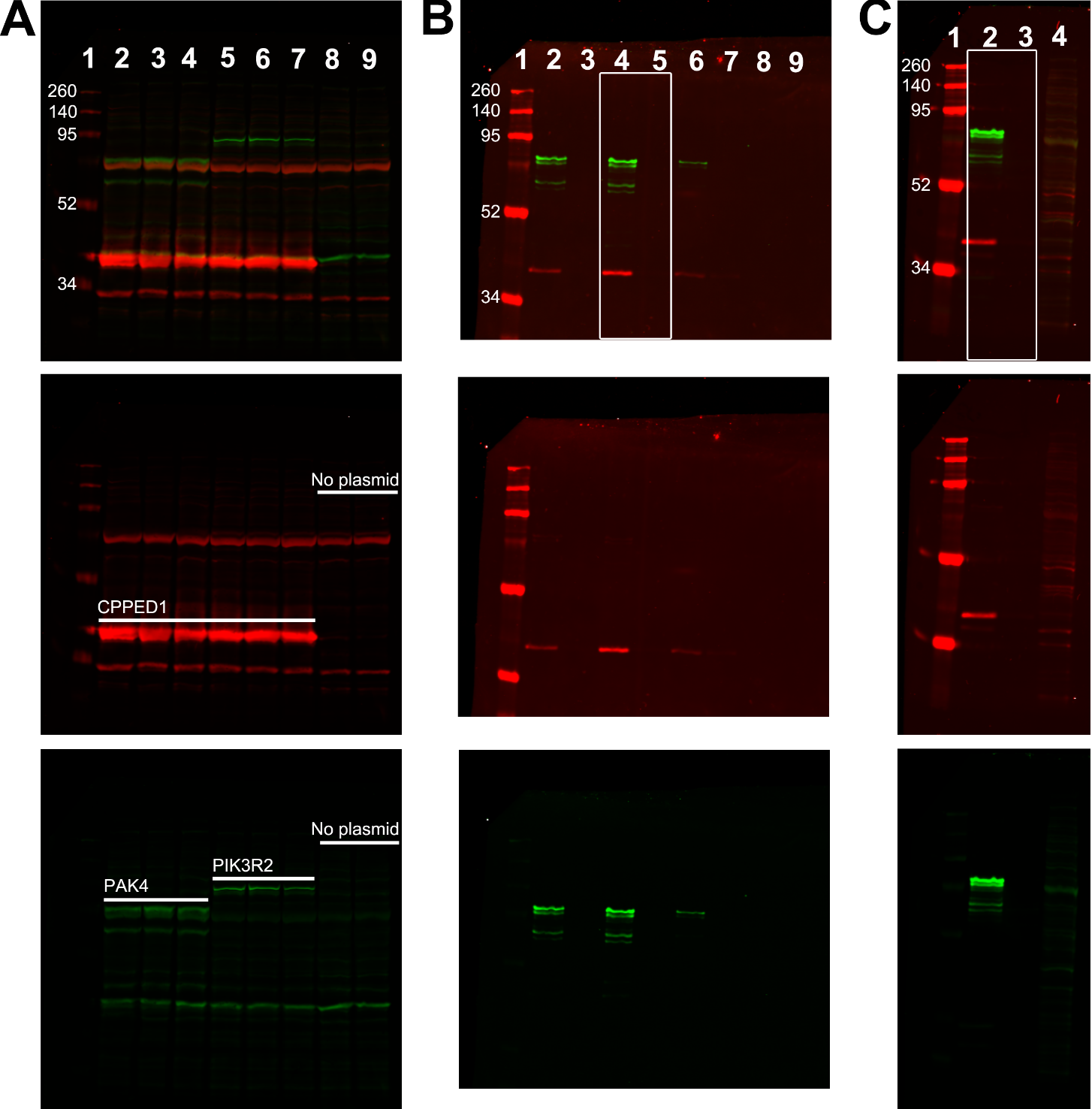


**Figure S8.** Coimmunoprecipitation of PAK4 with CPPED1. HEK293T cells expressing myc-PAK4, myc-PIK3R2, and flag-CPPED1 (A), coimmunoprecipitation of PAK4 with anti-myc Sepharose (B), and verification of the coimmunoprecipitated complex (C). Panel A shows, the following samples: 2–4, HEK293T cells co-transfected with CPPED1-pCMV-Flag 2B and PAK4-pcDNA3-myc (cell lysate, 40 µg); 5-7, HEK293T cells co-transfected with CPPED1-pCMV-Flag 2B and PIK3R2-pcDNA3-myc (cell lysate, 40 µg); and 8 and 9, untransfected HEK293T cells (cell lysate, 40 µg). In panel A, detection was done with rabbit anti-flag antibody (red, detecting flag-CPPED1), and mouse anti-myc antibody (green, detecting myc-PAK4 or myc-PIK3R2). Samples 2, 3, 4, and 8 from panel A were used to coimmunoprecipitate PAK4; results are shown in panel B. Myc-tagged PAK4 was coimmunoprecipitated with anti-myc Sepharose in three independent samples (2, 4, and 6; panel B). For the negative controls, anti-myc Sepharose was replaced by Sepharose (samples 3, 5, and 7; panel B). For an additional control, untransfected HEK293T cells (sample 8, panel A) were treated with anti-myc Sepharose (sample 8, panel B) or with Sepharose (sample 9, panel B) and processed the same way as all the other samples (samples 2–7, panel B). In panel B, detection was done with rabbit anti-flag antibody (red, detecting flag-CPPED1), and mouse anti-myc antibody (green, detecting myc-PAK4). Samples 4 and 5 from panel B were used to verify coimmunoprecipitated flag-tagged and myc-tagged proteins; results are shown in panel C. Panel C shows the following samples: 2, myc-tagged PAK4 coimmunoprecipitated with anti-myc Sepharose (sample 4 in panel B); 3, myc-tagged PAK4 coimmunoprecipitated with Sepharose (sample 5 in panel B); and 4, untransfected HEK293T cells (cell lysate, 20 µg). In panel C, detection was done with rabbit anti-CPPED1 antibody (red, detecting CPPED1), and mouse anti-PAK4 antibody (green, detecting PAK4). Cropped areas are shown in Fig. 3. Technical details are described in Materials and Methods (Coimmunoprecipitation).


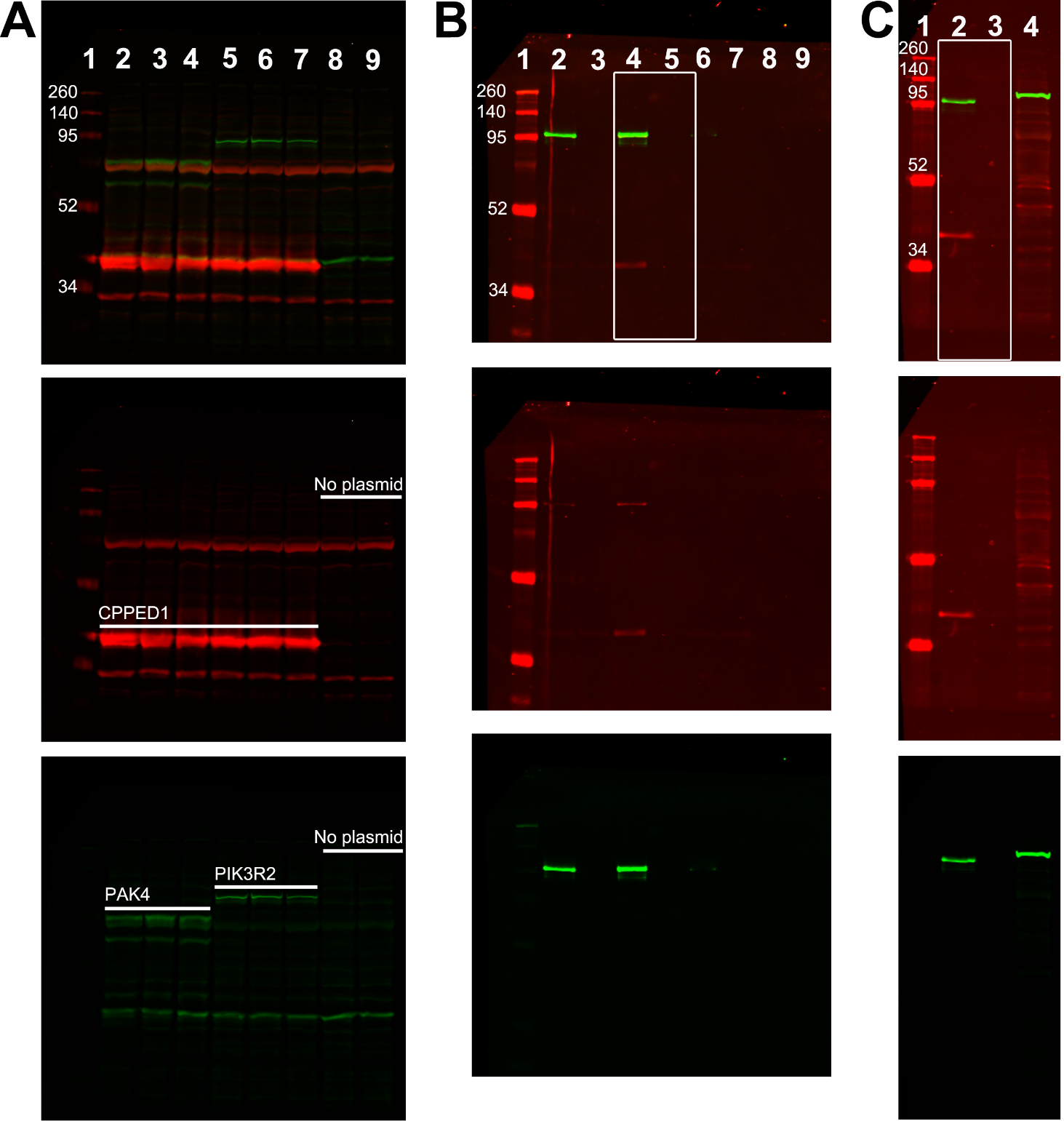


**Figure S9.** Coimmunoprecipitation of PIK3R2 with CPPED1. HEK293T cells expressing myc-PAK4, myc-PIK3R2, and flag-CPPED1 (A), coimmunoprecipitation of PIK3R2 with anti-myc Sepharose (B), and verification of the coimmunoprecipitated complex (C). Panel A shows the following samples: 2–4, HEK293T cells co-transfected with CPPED1-pCMV-Flag 2B and PAK4-pcDNA3-myc (cell lysate, 40 µg); 5–7, HEK293T cells co-transfected with CPPED1-pCMV-Flag 2B and PIK3R2-pcDNA3-myc (cell lysate, 40 µg); and 8 and 9, untransfected HEK293T cells (cell lysate, 40 µg). In panel A, detection was done with rabbit anti-flag antibody (red, detecting flag-CPPED1), and mouse anti-myc antibody (green, detecting myc-PAK4 or myc-PIK3R2). Samples 5–7 and 9 from panel A were used to coimmunoprecipitate PIK3R2; results are shown in panel B. Myc-tagged PIK3R2 was coimmunoprecipitated with anti-myc Sepharose in three independent samples (2, 4, and 6; panel B). For the negative controls, anti-myc Sepharose was replaced by Sepharose (3, 5, and 7; panel B). For an additional control, untransfected HEK293T cells (sample 9, panel A) were treated with anti-myc Sepharose (sample 8, panel B) or with Sepharose (sample 9, panel B) and processed the same way as all the other samples (samples 2–7, panel B). In panel B, detection was done with rabbit anti-flag antibody (red, detecting flag-CPPED1), and mouse anti-myc antibody (green, detecting myc-PIK3R2). Samples 4 and 5 from panel B were used to verify coimmunoprecipitated flag-tagged and myc-tagged proteins; the results are shown in panel C. In panel C, the following samples are shown: 2, myc-tagged PIK3R2 coimmunoprecipitated with anti-myc Sepharose (sample 4 in panel B); 3, myc-tagged PIK3R2 coimmunoprecipitated with Sepharose (sample 5 in panel B); and 4, untransfected HEK293T cells (cell lysate, 20 µg). In panel C, detection was done with rabbit anti-CPPED1 antibody (red, detecting CPPED1), and mouse anti-PIK3R2 antibody (green, detecting PIK3R2). Cropped areas are shown in Fig. 3. Technical details are described in the Materials and Methods (Coimmunoprecipitation).

**

**

**Figure S10.** Phosphopeptides of PAK4 determined by mass spectrometry. Amino acid sequence of human PAK4. Underlined sequences are phosphopeptides identified by mass spectrometry, and black dots indicate phosphorylated amino acid residues of the peptides. X indicates that any of these residues of the phosphopeptide were phosphorylated, but specific phosphorylated residue could not be determined. Black circles indicate amino acid residues with lower phosphorylation content when CPPED1 was included in activity measurements.

**

**

**Figure S11.** Phosphopeptides of PIK3R2 determined by mass spectrometry. Amino acid sequence of human PIK3R2. Underlined sequences are phosphopeptides identified by mass spectrometry, and black dots indicate phosphorylated amino acid residues of the peptides. X indicates that any of these residues of the phosphopeptide were phosphorylated but specific phosphorylated residue could not be determined. When CPPED1 was included in activity measurements, none of the phosphopeptides showed a decrease in their phosphate content.


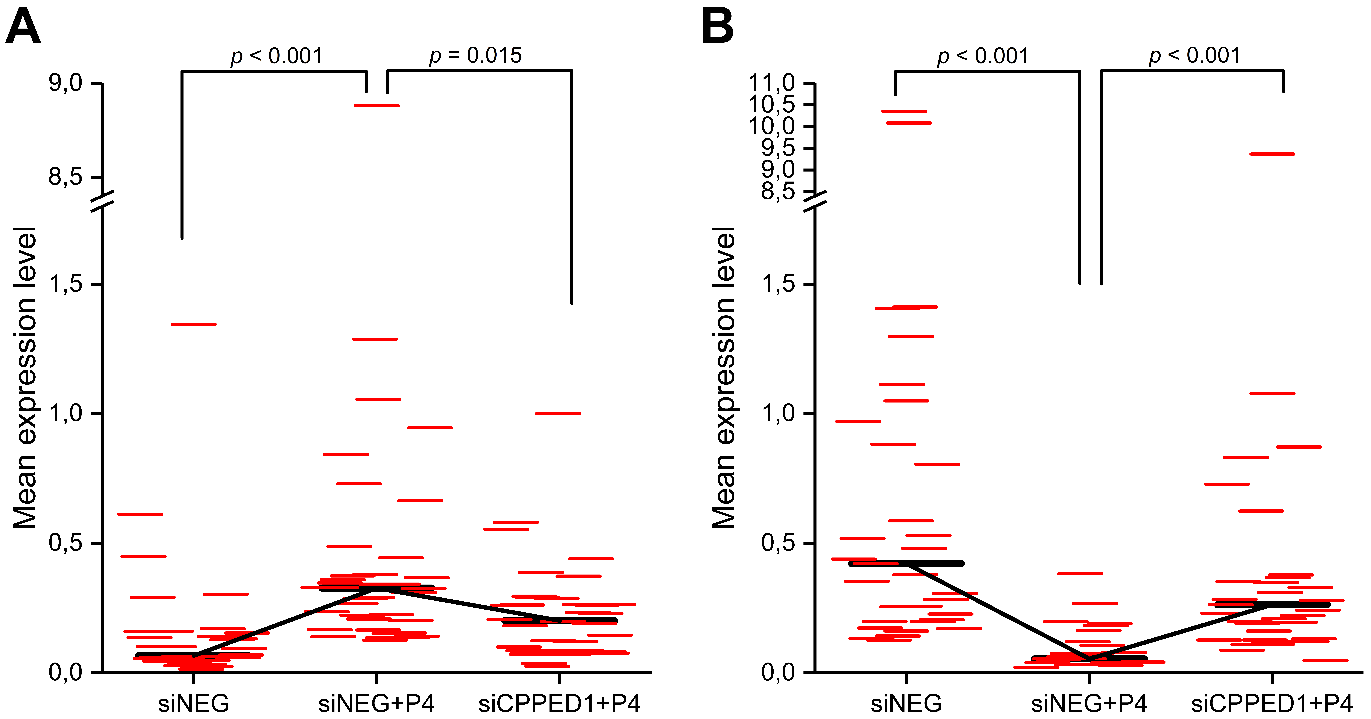


**Figure S12.** Mean expression levels of genes in HTR8/SVneo cells treated with siNEG, siNEG+P4 and siCPPED1+P4. The figure presents the data from Table S4. Transcriptomes of siNEG and progesterone (P4) treated siNEG (siNEG+P4) cells were compared to obtain a list of genes for which expression levels were affected by P4. P4 either increased (A, *n* = 38) or decreased (B, *n* = 33) the expression of genes. Only those genes for which the silencing of *CPPED1* expression removed the effect of P4 treatment are shown (red lines). Each red line represents normalized mean expression level values of a gene (three replicates). Differences were analyzed by nonparametric Kruskal–Wallis test. Horizontal line denotes the median of each group.


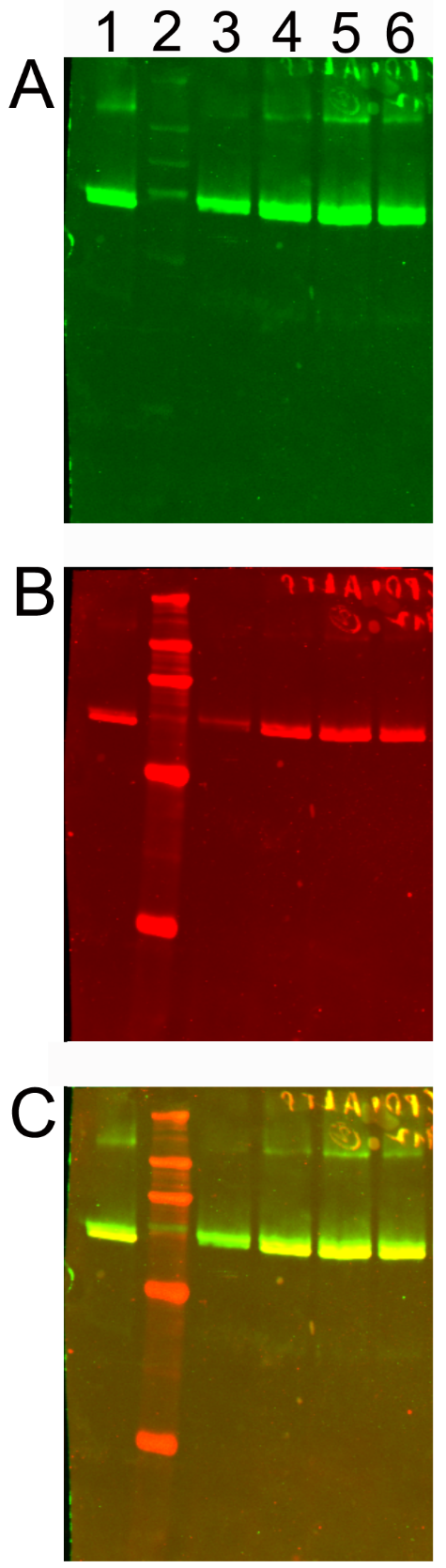


**Figure S13.** Representative image of detection of total AKT1 (A, green) and phosphorylated AKT1 (B, red) from the *in vitro* CPPED1 phosphatase assays (Fig. 1B). In panel C, A and B are overlaid. Technical details are described in the Materials and Methods (Detection of CPPED1 phosphatase activity by western blot). Commercially available recombinant His-tagged AKT1 was used as a run calibrator and positive control to detect both phosphorylated and unphosphorylated forms of AKT1. In this western blot, the following samples for the Fig. 1B data are shown: no CPPED1 (1), MnCl2 (3), CaCl2 (4), and no cation (5). Lane 2 is the molecular weight marker and, lane 6 contains 87 ng of recombinant His-tagged AKT1.
